# Comparative Proteomic Analyses Provide New Insights into Low Phosphorus Stress Responses in Maize Leaves

**DOI:** 10.1371/journal.pone.0098215

**Published:** 2014-05-23

**Authors:** Kewei Zhang, Hanhan Liu, Peilin Tao, Huan Chen

**Affiliations:** 1 School of Life Sciences, Shandong University, Ministry of Education Key Laboratory of Plant Cell Engineering and Germplasm Enhancement, Jinan, China; 2 College of Agriculture Vocational, Xuzhou Biology Engineering Technical College, Xuzhou, China; University of Antwerp, Belgium

## Abstract

Phosphorus deficiency limits plant growth and development. To better understand the mechanisms behind how maize responds to phosphate stress, we compared the proteome analysis results of two groups of maize leaves that were treated separately with 1,000 µM (control, +P) and 5 µM of KH_2_PO_4_ (intervention group, −P) for 25 days. In total, 1,342 protein spots were detected on 2-DE maps and 15.43% had changed (*P*<0.05; ≥1.5-fold) significantly in quantity between the +P and −P groups. These proteins are involved in several major metabolic pathways, including photosynthesis, carbohydrate metabolism, energy metabolism, secondary metabolism, signal transduction, protein synthesis, cell rescue and cell defense and virulence. The results showed that the reduction in photosynthesis under low phosphorus treatment was due to the down-regulation of the proteins involved in CO_2_ enrichment, the Calvin cycle and the electron transport system. Electron transport and photosynthesis restrictions resulted in a large accumulation of peroxides. Maize has developed many different reactive oxygen species (ROS) scavenging mechanisms to cope with low phosphorus stress, including up-regulating its antioxidant content and antioxidase activity. After being subjected to phosphorus stress over a long period, maize may increase its internal phosphorus utilization efficiency by altering photorespiration, starch synthesis and lipid composition. These results provide important information about how maize responds to low phosphorus stress.

## Introduction

Maize likely has the widest range of growing environments of all major crops. Maize has many uses, such as serving as a staple food and in biological and industrial applications [1]. Phosphorus is an indispensable macronutrient that plays a central role in plants, especially in photosynthesis [2]. Carbohydrates make up 95% of the dry weight of plants, and carbohydrate production relies on the rate of photosynthesis in leaves. Therefore, increasing the efficiency of photosynthesis would improve overall crop yields [3].

Physiological studies have demonstrated that plants respond to phosphorus deficiency in different ways, including improving phosphorus acquisition and internal phosphorus recycling. The mechanisms underlying these responses include intensified secretion of acid phosphatase [4], increased production of transcription factors [5] and phosphorus transporters [6] and altered root morphology [7]. In particular, some plant physiologists have suggested that phosphorus stress has several impacts on photosynthesis: (1) it affects energy transfer across the thylakoid membrane [8]; (2) it inactivates several pivotal enzymes involved in the Calvin cycle [9] and (3) there is feedback inhibition of photosynthesis across the thylakoid membrane through a reduction in electron transfer [10].

Gene expression profiles of maize, Arabidopsis, rice and soybean have revealed how phosphorus starvation affects plant growth and development [11]–[15]. The expression of certain genes encoding proteins involved in photosynthesis, including photosystems I (PSI) and II (PSII), ribulose-1,5-bisphosphate carboxylase/oxygenase (RuBisCO) and chlorophyll a/b-binding proteins, is repressed following phosphorus starvation [15]–[17]. The abundance of transcripts that encode products involved in sulfolipid biosynthesis, phospholipid degradation and starch biosynthesis increases to promote inorganic phosphate (Pi) utilization [6], [7]. Well over 100 genes that encode transcription factors and cell-signaling proteins are regulated during phosphorus starvation [11].

Proteome analysis by two-dimensional gel electrophoresis (2-DE) is a well-established technique that has been used to study a variety of plant responses to environmental stress [18]. Proteomic studies of winter rape roots, maize roots, *Arabidopsis thaliana* suspension cells, rice roots and leaves and soybean nodules have revealed biochemical changes in plants exposed to phosphorus starvation. Some of these changes are shared among tissues in different species, while some are unique [19]–[23]. Many proteins involved in hormone and organic acid synthesis are regulated to promote inorganic Pi absorption and mobilization [19], [23], [24]. Lan *et al.* found that *Arabidopsis thaliana* remodels the composition of lipid membranes and the activity of the glycolysis alternative pathway to increase internal phosphorus utilization efficiency during phosphorus deficiency [25]. Phosphorus starvation causes the accumulation of several defense- or stress-related proteins, such as superoxide dismutase (SOD), heat shock proteins (HSP) and proteins involved in the ubiquitin/26S proteasome pathway [20], [22].

Recently, proteomic analyses have begun to address the biochemical and molecular mechanisms behind the plant response to phosphorus deficiency. In this study, we analyzed the differential protein expression profiles of leaves using the inbred lines Qi319 to identify proteins that are differentially expressed under various phosphorus concentrations. This study provides valuable information that will lay the foundation for further studies of the functions of genes that respond to phosphorus deficiency.

## Materials and Methods

### Low phosphorus treatment and plant seedling growth

The seeds of the inbred maize line Qi319 were disinfected using 70% ethanol and HgCl_2_. They were then germinated in the dark at 28°C for 3 days, after which the seedlings (4 days old) were transferred to basic nutrient solution (1000 µM KH_2_PO_4_, +P) and grown until the 2–3 leaf stage. Then, half of the seedlings were transferred to low phosphorus nutrient solution (5 µM KH_2_PO_4_, -P) and the rest were allowed to continue growing in the +P nutrient solution for 25 days approximately to the 6–7 leaf stage [20], [26]. The composition of the basal nutrient solution (pH 6.0±0.1) was described previously [27]. Under low phosphorus conditions, the 1000 µM KH_2_PO_4_ in the +P nutrient solution was substituted with 1000 µM KCl. The nutrient solution was replaced every 3 days. The maize plants were grown at 25–30°C/18–20°C (day/night) with a 13.5 h light cycle (600–1200 µmol m^–2^ s^–1^). The relative humidity in the greenhouse was approximately 55–65%. The seedlings were positioned randomly in the greenhouse and three batches of seedlings were cultured separately, giving five experimental replicates in total.

### Physiochemical and proteome characteristics

#### Measurement of biomass, total plant phosphorus content and inorganic phosphorus concentration in leaves

The maize plants were harvested at the 6–7 leaf stage and washed twice with pure water. The shoots and roots were dried at 80°C to a constant weight and their weights were recorded respectively. The phosphorus concentration in the roots and shoots were determined according to Murphy *et al.* [28]. The inorganic phosphorus concentration in the shoots was determined according to Foyer *et al.* [8].

#### Measurement of chlorophyll (Chl), sucrose and starch concentrations in leaves

The Chl in the leaves was extracted using 95% ethanol and analyzed according to Arnon *et al.* [29]. Leaf samples were treated with resorcinol to measure the sucrose and starch concentrations [30].

#### Photosynthesis and chlorophyll fluorescence analysis

A portable photosynthesis system (LI-6400, LI-COR Inc., Lincoln, Nebraska, USA) was used to detect net photosynthesis (*Pn*), ambient carbon dioxide (*Co*), stomatal conductance (*Gn*), intercellular CO_2_ concentration (*Ci*) and transpiration rate (*Tr*) in the third expanded leaf. The stomatal limitation value (*Ls*) was then calculated using the formula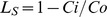. The photon flux density was kept at 800 µmol m^–2^ s^–1^ by an internal LED source; the temperature in the leaf chamber was maintained at 25°C and the relative air humidity was 55–60%. The CO_2_ concentration was approximately 400 µmol CO_2_ mol^−1^. All measurements were carried out between 09:30 h and 11:30 h.

After being exposed in the dark for 30 min, the maximum quantum efficiency of PSII photochemistry (*Fv/Fm*), photochemical efficiency of PSII in the light (*Fv’/Fm’*) photochemical quenching (*Qp*) and non-photochemical quenching (*NPQ*) were determined in the forth fully expanded leaves of the seedlings at room temperature using a pulse modulation chlorophyll fluorometer (FMS-2, Hansatech, UK). The allocation of photons absorbed by PSII was calculated by the formula according to Baker [31] as follows: the fraction of absorbed light in PSII antennae (*P*) utilized in PSII photochemistry from 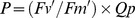; the fraction of absorbed light that dissipated thermally (*D*) was estimated from 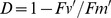; the fraction of light absorbed that dissipated other pathways (*E_X_*) was estimated from 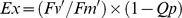 [32].

#### Enzyme Activity Assay

Fresh samples (1 g) were collected from the third leaves and ground rapidly in 4 ml of ice-pre-cooled buffer (0.1 mM Hepes–NaOH [pH 7.5]; 50 mM MgCl_2_; 2 mM EDTA; 2% PVP and 1% β-mercaptoethanol). The homogenates were centrifuged at 15,000×g for 20 min at 4°C and the supernatants were used for the enzyme assays [33]. For the fructose-1,6-bisphosphase (FBPase) assay, 30 mM Hepes-KOH (pH 8.2), 0.5 mm NADP^+^, 5 mM dithiothreitol (DTT), 5 mM MgCl_2_ and 2 to 4 units per mL of phosphoglucose isomerase and glucose-6-phosphorus dehydrogenase were used. The reaction was initiated by the addition of 5 mM fructose-1,6-bisphosphate (FBP) [33]. RuBisCO activity was assayed by monitoring the absorbance change at 340 nm due to the oxidation of NADH (*ε* = 6.22 mM^−1^ cm^−1^) according to the method of Sawada *et al.* [34]. For the ascorbate peroxidase (APX) activity assay, the leaf tissue samples were ground in 5 ml buffer (0.05 mM phosphate buffer [pH 7.8], 0.1 mM EDTA) and centrifuged at 12,000×g for 15 min at 4°C. The supernatants were kept on ice for the enzyme assay. The APX activity was assayed by detecting the shift in ascorbate oxidation at 290 nm (ε = 2.8 mM^−1^ cm^−1^) [35].

#### Measurement of leaf ascorbic acid (ASA), H_2_O_2_, malondialdehyde (MDA), ATP contents, ion leakage and the rate of O_2_^. –^ production

For the O_2_^. −^ production rate assay, 1.0 g of leaf tissue was ground in 4 ml of buffer (0.1 mM phosphate buffer [pH 7.8], 0.1 mM EDTA, 4% w/v PVPP and 0.3% Triton X-100) and after that centrifuged at 12,000×g at 4°C for 20 min. The supernatants were kept on ice for assaying. The O_2_^. −^ production rate was determined according to Wang *et al.* [36].

The ASA content was assayed by monitoring the rate of osazone production. Fresh leaf samples (0.5 g) were ground in 10 ml 6% trichloroacetic acid (TCA) and centrifuged at 12,000×g for 20 min at 4°C. Then, 4.0 ml of the supernatant was added to 2 ml of dinitrophenylhydrazine (in sulfuric acid) containing one drop of thiourea (in 70% ethanol). The mixture was boiled for 15 min in a water bath. After cooling, 5 ml of 80% (v/v) H_2_SO_4_ was added to the supernatant. The absorbance at 530 nm was measured [37].

The H_2_O_2_ concentration was measured by determining the amount of titanium hydro-peroxide complex produced according to the method of Mukherjee *et al.* [38]. Fresh leaf samples (1 g) were ground with a mortar and pestle in 5 ml of ice-cold acetone and centrifuged at 3,000×g for 10 min at 4°C, followed by 5,000×g for 20 min at 4°C. Then, titanous sulfate and aqueous ammonia were added. The precipitate that came from the reaction with titanium sulfate and ammonia was dissolved in 5 ml H_2_SO_4_ (2M) and the absorption was measured at 415 nm.

The ATP concentration in maize leaves was assayed by the method described by Fan *et al.* [39]. ATP content was determined by fluorescence intensity, which was measured with a F4500 Fluorescence Spectrophotometer. Luciferase catalyzes the fluorescence of the substrate at an intensity related to the ATP concentration. Specifically, 1.0 g fresh samples were boiled with 5 ml 0.5 mol/L MgSO_4_ for 15 min, and the solution was centrifuged at 5,000×g for 15 min at 4°C. The supernatant was incubated on ice. Then, 0.2 ml supernatant was added to 0.8 ml relevant buffer and the fluorescence intensity of the mixture was recorded to calculate the ATP concentration based on an ATP standard curve.

The MDA levels of leaves were assayed according to Quan *et al.* as follows [40]. Fresh leaf samples (200 mg) were homogenized in 5 ml of 10% TCA and centrifuged at 12,000×g for 10 min at 4°C. Two milliliters of the supernatant was added to 4 ml of 0.6% thiobarbituric acid and the reaction mixture was incubated in boiling water for 15 min. The reaction was terminated by cooling in an ice bath. The absorbance of the supernatant at 450, 532 and 600 nm was detected with a spectrometer. The MDA concentration was calculated by the following formula:
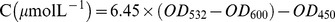



The ion leakage from the maize leaf cellular membranes under phosphorus deficiency was determined by conductivity measurement of electrolyte leakage from the leaves. Detached leaves of approximately 100 mg were washed three times with redistilled water and blotted onto filter paper. After that the leaves were cut into several pieces and placed into 25 ml of redistilled water. The samples were vacuumized to 0.05 MPa for 20 min and incubated at 25°C for 2 h, and the ion leakage of the samples was measured with a conductivity meter. The ion leakage was expressed as a percentage and was calculated as described by Lv *et al.* [41].

#### Measurement of photorespiration rate

The photorespiration rate was measured using an LI-6400/XT System according to the LI-COR News Line (http://www.licor.com/env/newsline/2011/02/ measuring-photorespiration-with-the-li-6400xt-system/). A tank of air with 2% oxygen and a tank of normal air with 21% oxygen with were supplied to the LI-6400/XT System for the photosynthesis light source respectively, and the net photosynthesis (*Pn*) was detected. In addition, the photosynthetically active radiation (*PAR*) was set to 1200 µmol m^−2^ s^−1^ and 380 µmol CO_2_ mol^−1^. The D-value between the two results represents the photorespiration rate.

All physiological data were presented as the mean ± SD. Variance analysis between +P and -P maize plants was performed using SPSS 16.0 software.

#### Protein sample preparation

For each sample, tissue from the middle of the third leaf (2 g) was ground to a powder in liquid nitrogen and resuspended in Mg^2+^/CHAPS buffer (0.5 M Tris-HCl, 2% CHAPS, 20 mM MgCl_2_, 10 mM DTT, 1 mM PMSF). The sample was centrifuged at 19,000×g for 20 min at 4°C. The supernatant was then subjected to further fractionation with 12.5% PEG [42]–[44]. The pellet and supernatant were suspended once more in TCA/acetone solution (contain 10 mM DTT, 1 mM PMSF) and precipitated at −20°C. The mixture was then centrifuged at 19,000×g at 4°C for 20 min. The pellet was carefully washed twice in acetone (contain 10 mM DTT, 1 mM PMSF) to remove any pigment [45] and vacuumized with a Speed-Vac. The samples were then dissolved separately in protein solubilization buffer (7 M urea, 2 M thiourea, 4% CHAPS, 0.5% v/v carrier ampholyte, pH 3–10, 10 mM DTT, 1 mM PMSF) for 2 h. The insoluble material was removed by centrifugation at 40,000×g for 25 min. The protein concentration in the supernatant was then measured using the Bradford assay and sub-packaged for 2-DE analysis [46].

#### 2-DE mapping and image analysis

2-DE was performed using pH 5–8 immobilized pH gradient (IPG) strips (Bio-Rad). A liquid rehydration buffer containing 1.5 mg protein (7 M urea, 2 M thiourea, 4% CHAPS, 1.5% v/v carrier ampholyte, 65 mM DTT) was used to hydrate the strips for 13 h using a GE Healthcare III spectrophotometer. The voltage procedure was as follows: (1) Grade voltage increased to 250 V for 1 h; (2) Grade voltage increased to 1,000 V for 1 h; (3) Step voltage to 5,000 V for 3 h; (4) Grade voltage increased to 10,000 V for 6 h and (5) Step voltage increased to 10,000 V and the focus increased to 100 kVh. After isoelectric focusing, the strips were equilibrated prior to SDS-PAGE according to the methods of Zhao *et al.* [47]. The strips were loaded onto 12% denaturing acrylamide gels and sealed with 0.5% agarose solution. The electrophoresis was carried out using a PROTEANII Ready Gel System (20 cm×20 cm, Bio-Rad) at 10 mA/gel for 1 h and 25 mA/gel for 6 h. The gels were stained with coomassie brilliant blue (CBB) according to Katam *et al.* [48] and scanned using a GS-800 Calibrated Densitometer (Bio-Rad). The images were analyzed using PDQuest software (version 7.2.0; Bio-Rad). After background subtraction and spot detection, the spots were matched and normalized using the method of total density in the gel images. The statistical significance of the quantitative data was determined by Student's *t*-test (n = 3, P<0.05) at a 95% confidence level. Where the identified proteins showed a 1.5-fold or more change in concentration between the two phosphorus concentration treatments, they were considered to have differentially accumulated between the –P and +P treatments.

#### In-gel digestion and MALDI-TOF/MS and MALDI-TOF-TOF analysis

Several protein spots were excised from the gels and washed twice with distilled water to remove the redundant sodium dodecyl sulfonate (SDS). The excised gel pieces were then destained using 25 mM NH_4_HCO_3_ and dehydrated with 100% acetonitrile (ACN). The protein spots were reduced, alkylated and washed thoroughly, according to Yan *et al.* [49], followed by digestion with 5–8 µl trypsin (proteome grade, Sigma) for 30 min at 4°C. Next, the redundant enzyme was removed and the spots were covered with 15 µl 25 mM NH_4_HCO_3_ solution (pH 8.0) and incubated overnight. The supernatant was then transferred to a new centrifuge tube and 25 µl of 67% ACN, 3.3% trifluoroacetic acid (TFA) solution was added. The two supernatant liquids were combined and dried in a Speed-Vac, dissolved in 4–5 µl 0.1% TFA and stored in 0.5 µl aliquots at −80°C. In preparation for analysis, the samples were mixed with 0.6 µl 10 mg ml^−1^ w/v alpha reached-4-hydroxylcinnamic acid (CHCA, Sigma) in 0.1% TFA/50% ACN in the metal plate. After being air-dried, the samples were subjected to MALDI-TOF/MS and MALDI-TOF-TOF, which were controlled by the Flexcontrol 2.4 package using default parameters (Bruker Daltonics, Karlsruhe, Germany).

#### Protein identification and database searching

After the data were calibrated and picked, monoisotopic peak analysis was conducted using GPS Explorer (Applied Biosystems 2006) and the monoisotopic peak lists were used for analysis with the Mascot program (http://www. matrixscience.com) against NCBInr (non redundant national center for biotechnology information database), allowing one mistake trypsin miscleavage. Carbamidomethylation of Cys and oxidation of the Met were recognized as the fixed modifications, with pyro-Glu formation of N-terminal Gln as the variable modification. To obtain high confidence identification results, the protein had to fulfill the following criteria: (1) the MOWSE score was not below 72 (P<0.05); (2) more than six peptides matched the theoretical results; (3) the protein must be selected from the first or second database reports; (4) the Sequence Coverage must be more than 15% and (5) 0.3 Da peptide mass tolerance. The search criteria for the MALDI-TOF-TOF results were similar to peptide mass fingerprinting (PMF): (1) the individual ions scores >43 (P<0.05); (2) the Peptide Mass Tolerance was 100 ppm and (3) Fragment Mass Tolerance was 0.3 Da. The proteins identified using MALDI-TOF/MS were categorized using the MIPS *Arabidopsis thaliana* Database (MATDB) database (http://mips.gsf.de/proj/thal/db/) and their function (in terms of metabolic and regulatory pathways) was further analyzed.

## Results

### Maize leaf growth and physiological responses to phosphorus stress

**Figure 1:**
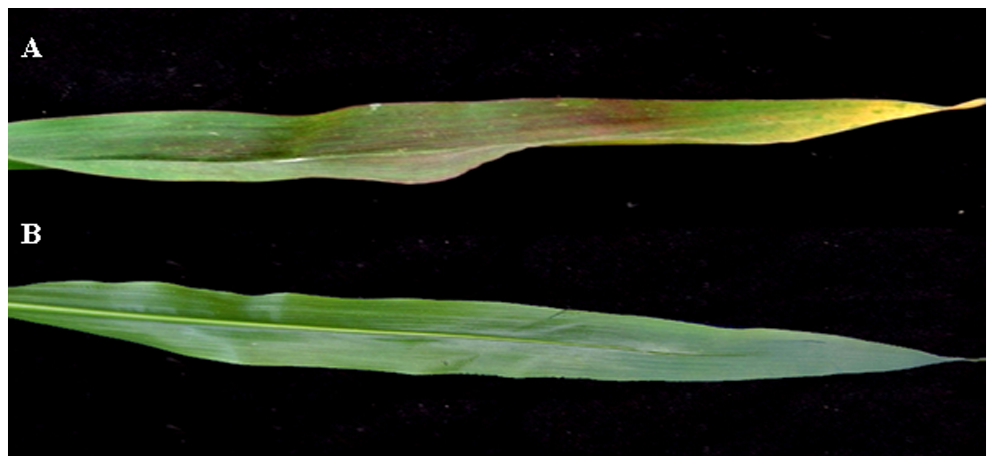
The fourth leaf of maize plants treated with low phosphorus solution for 25 d. A, treatment with 5 µmol phosphorus; B, treatment with 1,000 µmol phosphorus.

After treatment with 5 µmol phosphorus for 25 days, the maize leaves displayed apparent phosphorus deficiency symptoms, including restricted growth, a decline in phosphorus concentration, reduced inorganic phosphorus contents and marked changes in biomass (Table 1). The leaves of maize under low phosphorus conditions displayed heliotrope-colored stems, and the leaf tips were withered and yellow when treated with 5 µmol KH_2_PO_4_ (Figure 1A). Maize plants under 1000 µmol KH_2_PO_4_ treatment had dark-green leaves (Figure 1B).

**Table 1:** Influence of phosphorus starvation on plant biomass, phosphorus content and chlorophyll, inorganic phosphorus and ATP concentration.

Pi treatment	Plant biomass (g Dw /plants)	Chlorophyll concentration (mg*g^−1^ Fw)	Phosphorus content (mg P/plant)	Inorganic phosphorus concentration (µg*g^−1^ Fw)	ATP concentration (nmol*g^−1^ Fw)
+P	5.94±0.11	1.73±0.05	17.48±1.17	18.10±0.84	565.45±25.34
-P	3.11±0.13*	0.92±0.05*	2.94±0.12*	8.36±0.44*	399.65±6.78*

The reported values represent the mean of 15 seedlings ± SD. * indicates significant difference at p<0.05 compared with +P plants.

Low phosphorus levels decreased the net photosynthetic rate (*Pn*) and stomatal conductance (*Gs*) but increased the intercellular CO_2_ concentration (*Ci*) (Table 2). The phosphorus content within cells may affect the proportional distribution of derived carbon between sucrose and starch. In this study, the sucrose content in the leaves declined as the starch content increased, and the ratio between sucrose and starch (sucrose/starch) also decreased under low phosphorus conditions. In addition, the activity of FBPase, which is involved in the sucrose synthesis pathway, also declined under phosphorus deficient conditions (Table 3). Photorespiration is one of the subsidiary reactions of photosynthesis. The photorespiration data showed that photorespiration increased under low phosphorus stress by 57.75% (Table 3). In addition, the chlorophyll and ATP concentrations in the leaves were noticeably reduced (Table 1).

**Table 2:** Influence of phosphorus starvation on *Pn*, *Ci*, *Gs*, *Ls* and RuBisCO activity.

Pi treatment	*Pn* (µmol CO_2_ m^−2^ s^−1^)	*Ci* (µmol CO_2_ m^−2^ s^−1^)	RuBisCO (µmolCO_2_ *mg pr^−1^min^−1^)	*Gs* (mmol H_2_O m-^2^s^−1^)	*Ls*
+P	18.83±0.40	149.51±3.17	0.41±0.02	147.17±2.80	0.63±0.06
-P	7.40±0.68*	211.68±5.12*	0.27±0.01*	88.96±2.35*	0.52±0.02*

The reported values represent the mean of 15 seedlings ± SD. * represents significant difference at p<0.05 compared with +P plants.

**Table 3:** Influence of phosphorus deficiency on sucrose and starch concentration, photorespiration rate and FBPase activity in leaves.

Pi treatment	Sucrose (mg*g^−1^ Dw)	Starch (mg*g^−1^Dw)	Sucrose/Starch	FBPase activity (µmolNADPH*mg^−1^ pr*min^−1^)	Photorespiration rate(µmolCO_2_*m^−2^* s^−1^)
+P	37.91±1.10	183.01±1.85	0.21±0.0078	0.91±0.02	0.71±0.071
-P	27.30±0.84 *	206.93±1.64*	0.13±0.0039 *	0.81±0.03*	1.12±0.12*

The reported values represent the mean of 15 seedlings ± S.D. * represents significant difference at p<0.05 compared with +P plants.

Abiotic stress may affect the balance of reactive oxygen species (ROS) homeostasis in leaves. The physiological data showed that the O_2_^−^ production rate, the levels of ion-leakage, H_2_O_2_, MDA and ASA, and APX activity increased significantly under phosphorus-deficient conditions (Table 4).

**Table 4:** Influence of phosphorus deficiency on the rate of O_2_^. −^ production, the concentration of H_2_O_2,_ MDA and ascorbic acid, ion-leakage and APX activity.

Pi treatment	O_2_^−^ production rate (nmol*min^−1^mg^−1^Pr)	H_2_O_2_ concentration (µmol*g^−1^Fw)	Ion leakage (%)	MDA (nmol*g^−1^Fw)	Ascorbic Acid concentration (mg*g^−1^Fw)	APX activity (U*mg^−1^Pr)
+P	0.115±0.014	0.35±0.05	16.41±1.61	24.16±1.11	0.35±0.07	35.35±0.78
-P	0.194±0.026*	0.84±0.08*	25.64±1.68*	32.34±1.73*	0.73±0.02*	47.17±1.21*

The reported values represent the mean of 15 seedlings ± S.D. * represents significant difference at p<0.05 compared with +P plants.

*Fv/Fm* is used to indicate the maximum light absorption efficiency by PSII. *Fv/Fm* decreased by 14.86% under low phosphorus stress compared with the sufficient phosphorus treatment. The absorption of photon energy in the maize leaf is divided into three parts: the fraction of light absorbed in PII that is used for photochemical reactions in PSII (*P*); the fraction of light absorbed in PSII that is lost through heat dissipation by the antenna complex (*D*) and the fraction of light absorbed in PSII that is not dissipated by *P* and *D* but is dissipated by other pathways, such as the xanthophyll cycle and active oxygen scavenging as excess energy (*Ex*). Compared to the sufficient phosphorus treatment, under phosphorus starvation, *D* and *Ex* increased by 10.53% and 20.83%, respectively, whereas *P* decreased by 57.89%. These results, combined with the PSII actual quantum efficiency (*ΦPSII*), *qP* and *NPQ* data, demonstrate that the photon energy distribution is altered by phosphorus deficiency stress (Table 5).

**Table 5:** Influence of phosphorus deficiency on chlorophyll fluorescence parameters.

Pi treatment	*Fv/Fm*	*qP*	*NPQ*	*D*	*P*	*Ex*
+P	0.74±0.01	0.41±0.02	2.07±0.03	0.57±0.04	0.19±0.03	0.24±0.07
-P	0.63±0.01*	0.26±0.03*	2.42±0.05*	0.63±0.06*	0.08±0.03*	0.29±0.02*

The reported values represent the mean of 15 seedlings ± S.D. * represents significant difference at p<0.05 compared with +P plants.

### The proteome profile of maize leaves

**Figure 2:**
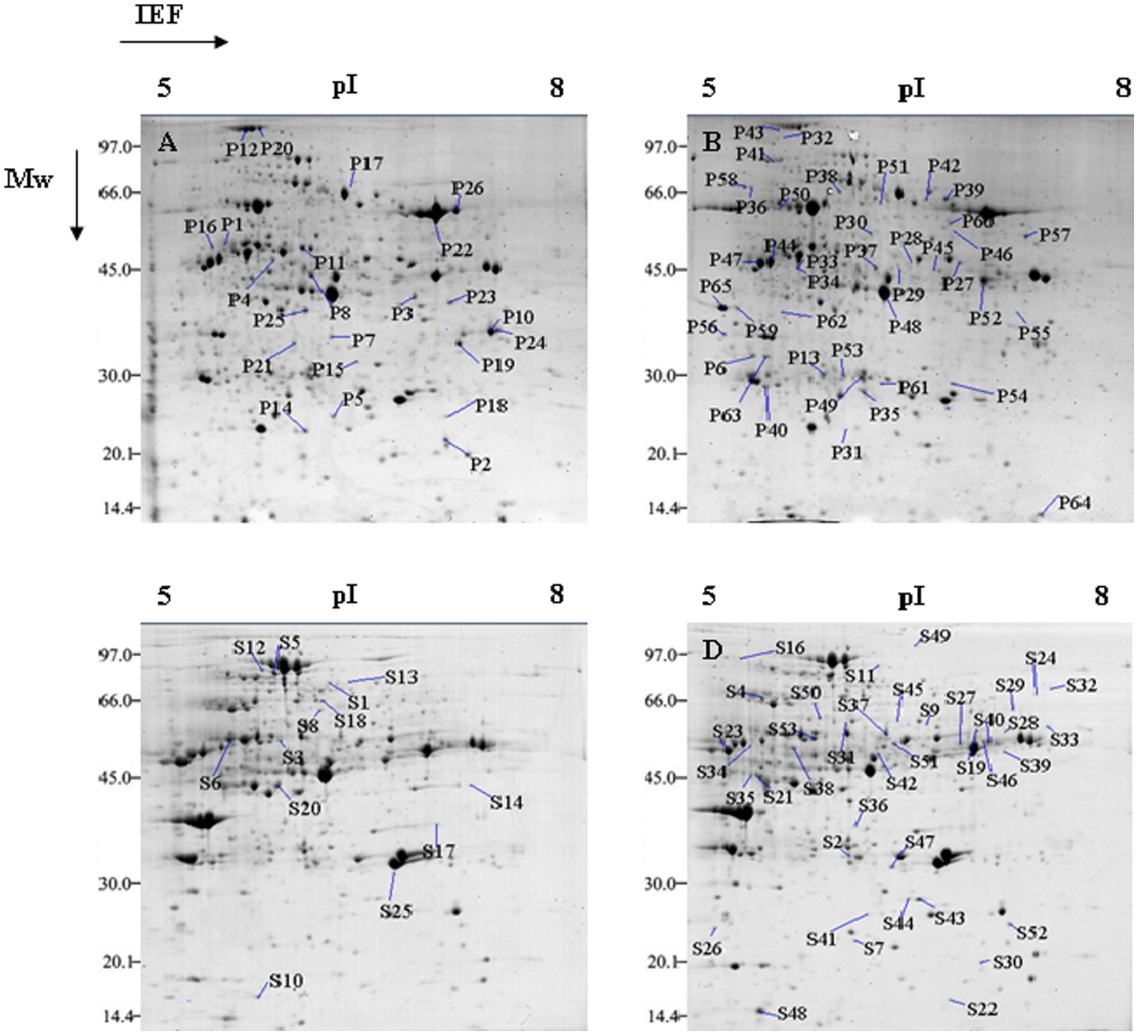
Comparison of 2-DE gel maps of proteins from maize leaves. The proteins were extracted using a PEG fractionation technique. The 1.5(IEF) using 17 cm pH 5–8 IPG strips, then placed on a 12% polyacrylamide gel for the second dimensional separation and stained with CBB. The gel image analysis was carried out using PDQuest software (version 7.2.0; BioRad). The spots marked with numbers were identified by MALDI-TOF MS. A: image of +P treatment pellet (P) proteins; B: image of –P treatment pellet (P) proteins; C: image of +P treatment supernatant (S) proteins; D: image of –P treatment supernatant (S) proteins.

Comparative proteomic studies were carried out using IPG strips (pH 5–8) on Qi319 maize leaves that had been subjected to different phosphorus concentrations treatments. The results showed that 750 spots were detected when the +P treatment solid pellet was analyzed. Of these, 100 spots (13.3%) had changed significantly compared to the same spots observed when the low phosphorus treatment pellet was analyzed (*P*<0.05). These differentially expressed proteins included 46 spots whose size increased (including newly appearing spots) and 54 spots whose size decreased (including spots that had disappeared; Figure 2A, B). In the supernatant liquid, 100 (16.89%) of the 592 spots detected had changed significantly under +P treatment (*P*<0.05), including 33 spots that showed an increasing size pattern (including newly appearing spots) and 67 spots whose size had decreased (including spots that had disappeared) compared to the low phosphorus treatment gels (Figure 2C, D). In summary, approximately 1342 spots were stained by CBB and 200 spots (14.91%) showed an obvious difference between the +P and −P treatments. These results indicate that phosphorus-deficient conditions results in major proteomic changes in maize leaves.

### Identification and classification of proteins involved in maize leaf responses to phosphorus deficiency

We identified the differentially expressed proteins by MALDI-TOF/MS to gain a better understanding of the mechanisms used by maize to respond to phosphorus deficiency. We identified 116 proteins based on Mascot and NCBInr (Table 6 and Table S1). These 116 proteins were classified based on MATDB (http://mips.sf.de/proj/thal/db/, http://mips.gsf.de/proj/thal/db/). The classifications were separated into protein fate, protein synthesis, cell rescue/defense/virulence, metabolism, energy, transcription/cellular communication/signal transduction mechanisms and cell cycle/transport (Table 6). To confirm the results of peptide mass fingerprinting (PMF), 15 spots were randomly selected from the proteins identified by PMF and subjected to MALDI-TOF-TOF. The results from 14 of the spots were consistent with the PMF results (Table 7 and Text S1). This result confirmed the reliability of the PMF results.

**Table 6:** Identification of differentially expressed proteins in leaves of maize treated with 5 µmol/L KH_2_PO_4_ and 1,000µmol/L KH_2_PO_4_ for 25 days.

SPOT NO *^a^*	Protein name *^b^*	PlantSpecies *^c^*	gi Number *^d^*	Theoretical pI *^e^*	Experimental pI *^f^*	Experimental Mw*^g^*	Theoretical Mw *^h^*	SC% *^i^*	Score *^k^*	Specify *^m^*	QM *^n^*
metabolism											
P1	ATP synthase beta subunit	*Bouteloua curtipendula*	gi|150035719	5.52	5.57	50.9	46.7	57	96	7.97∶1	15
P2	ribulose-1,5-bisphosphate carboxylase/oxygenase large subunit	*Sorghastrum nutans*	gi|144583486	6.34	7.01	24.2	52.9	16	75	7.18∶1	10
P4	ribulose bisphosphate carboxylase/oxygenase activase, chloroplastic precursor	Zea mays *Zea mays*	gi|162458161	6.29	5.88	48.4	48.1	37	84	3.42∶1	16
P7	ATP synthase subunit gamma, chloroplastic	*Zea mays*	gi|110278822	8.44	6.27	36.1	40.1	29	165	1∶0	16
P8	fructose-1,6-bisphosphatase	*Zea mays*	gi|194703704	5.59	7.28	41.5	38.1	36	76	3.00∶1	10
P10	ribulose-1,5-bisphosphate carboxylase/oxygenase large subunit	*Zea mays*	gi|144583510	6.57	7.31	35.1	46.9	37	208	2.62∶1	27
P11	glutamine synthetase	*Zea mays*	gi|195619796	5.5	6.08	47.9	42.1	29	78	2.57∶1	9
P12	pyruvate, orthophosphate dikinase	*Zea mays*	gi|168586	5.71	5.68	109.8	103.4	29	160	2.45∶1	25
P14	ribulose-1,5-bisphosphate carboxylase/oxygenase large subunit	*Calophyllum membranaceum*	gi|331704204	6.19	6.11	22.8	19.4	28	77	2.24∶1	6
P18	phosphoenolpyruvate carboxylase	*Zea mays*	gi|194703414	8.87	7.03	26.4	39.1	24	80	1∶0	8
P19	ribulose-1,5-bisphosphate carboxylase/oxygenase large subunit	*Sorghastrum nutans*	gi|144583486	6.34	7.1	33.8	52.9	31	179	3.28∶1	23
P20	pyruvate, orthophosphate dikinase	*Zea mays*	gi|168586	5.71	5.79	117.1	103.4	19	108	1.97∶1	21
P21	esterase-like protein	*Chlamydomonas* reinhardtii	gi|159481289	9.16	6.13	35.2	35.3	15	77	1.89∶1	7
P22	ribulose-1,5-bisphosphate carboxylase/oxygenase large subunit	Saccharum hybrid cultivar SP-80-3280	gi|48478779	6.33	6.69	58.9	53.3	28	107	3.74∶1	15
P23	ribulose-1,5-bisphosphate carboxylase/oxygenase large subunit	*Zea mays*	gi|11467200	6.58	6.33	53.3	50.6	29	136	2.79∶1	15
P24	ribulose-1,5-bisphosphate carboxylase/oxygenase large subunit	*Parodiophyllochloa ovulifera*	gi|164565071	6.44	7.34	35.2	49.9	21	74	2.20∶1	13
P25	NADP-dependent oxidoreductase P1	*Zea mays*	gi|226528403	6.23	6.1	39.8	39.2	43	109	18.82∶1	12
P26	ribulose-1,5-bisphosphate carboxylase/oxygenase large subunit	*Miscanthus capensis*	gi|125991663	6.46	7.08	61.1	49.9	38	171	2.84∶1	24
P33	succinyl-CoA synthetase beta subunit	*Zea mays*	gi|226510248	5.99	5.74	43.5	45.5	26	73	0∶1	9
P34	glycerophosphodiester phosphodiesterase	*Zea mays*	gi|195619594	5.28	5.74	42.9	41.7	20	79	0∶1	8
P37	alpha-1,4-glucan-protein synthase	*Zea mays*	gi|162463414	5.75	6.24	42.9	41.7	37	103	1∶1.63	12
P38	ketol-acid reductoisomerase	*Sorghum bicolor*	gi|242091437	5.88	6	65.1	63.6	19	75	1∶2.98	11
P42	beta-D-glucosidase precursor	*Zea mays*	gi|343227637	6.75	6.54	65.2	63.5	22	78	1∶3.09	11
P43	lipoxygenase	*Zea mays*	gi|93211180	6.11	5.63	90.7	102.4	20	83	1∶2.32	12
P46	GDP-D-mannose-3',5'-epimerase	*Zea mays*	gi|212275446	5.99	6.73	53.6	43.3	30	82	0∶1	10
P47	phosphoribulokinase	*Zea mays*	gi|195645472	5.75	5.51	45.9	46.1	26	81	1∶2.69	13
P51	adenosylhomocysteinase	*Zea mays*	gi|226491362	5.63	6.26	61.9	53.9	25	80	1∶4.05	11
P52	NADP-dependent oxidoreductase P1	*Zea mays*	gi|226528403	6.23	6.93	43.8	39.2	43	109	0∶1	12
P57	UDP-sulfoquinovose synthase	*Zea mays*	gi|226530866	8.39	7.21	54.8	53.1	31	100	1∶6.86	12
P64	nucleoside diphosphate kinase Group I	*Zea mays*	gi|293332239	6.84	7.28	12.1	16.8	48	84	1∶2.00	9
S1	ferredoxin—nitrite reductase, chloroplastic	*Zea mays*	gi|128350	6.05	6.25	70.3	63.8	23	92	1.91∶1	10
S5	diacylglycerol kinase	*Zea mays*	gi|11994267	6.22	5.88	75.1	54.4	15	74	2.46∶1	10
S6	NADP-dependant malate dehydrogenase	*Zea mays*	gi|334085713	5.05	5.58	48.6	36.7	42	107	2.60∶1	11
S8	sucrose-phosphatase 1	*Zea mays*	gi|162463022	5.48	6.19	63.5	47.6	27	87	3.29∶1	9
S10	transketolase chloroplastic	*Zea mays*	gi|194708072	5.46	6.02	18.1	68.5	17	74	6.47∶1	11
S12	phosphoglucomutase, cytoplasmic 1	*Zea mays*	gi|162463106	5.46	5.8	77.8	63.3	31	98	8.63∶1	15
S13	ferredoxin—nitrite reductase chloroplastic	*Zea mays*	gi|195615808	6.47	6.37	70.3	66.7	34	134	1∶0	16
S18	threonine synthase	*Zea mays*	gi|226504054	6.44	6.2	66.2	58. 1	37	153	2.29∶1	17
S19	formate dehydrogenase 1	*Zea mays*	gi|195640660	6.32	6.89	53.7	41.7	34	74	1∶1.99	10
S23	phosphoribulokinase	*Zea mays*	gi|226498182	5.84	5.24	54.3	45.1	18	73	1∶2.43	9
S24	alanine aminotransferase 2	*Zea mays*	gi|226531608	6.73	7.32	66.4	53.7	38	125	1∶3.40	16
S25	chloroplast light-harvesting complex I protein precursor Lhca3	*Acetabularia acetabulum*	gi|145079211	5.73	6.66	30.4	22.2	26	75	3.76∶1	6
S29	UDP-sulfoquinovose synthase	*Zea mays*	gi|238014584	8.39	7.15	61.3	53.0	30	82	1∶8.70	10
S32	serine hydroxymethyltransferase	*Zea mays*	gi|195622500	6.84	7.41	69.2	52.1	28	78	1∶6.68	9
S33	phosphoserine aminotransferase	*Zea mays*	gi|194701280	8.55	7.39	55.4	45.2	34	108	1∶5.54	9
S45	GDP-mannose 3,5-epimerase 1	*Zea mays*	gi|195620882	5.94	6.4	59.8	43.3	42	102	1∶3.44	13
Protein synthesis											
P5	50S ribosomal protein L10	*Zea mays*	gi|226529501	8.86	6.27	26.6	26.6	56	107	5.28∶1	10
P16	elongation factor Tu	*Zea mays*	gi|226508704	6.07	5.48	51.6	50.8	38	108	4.11∶1	17
P17	asparaginyl-tRNA synthetase, cytoplasmic 3	*Zea mays*	gi|226507460	6.05	6.39	64.8	63.5	40	170	2.01∶1	22
P32	elongation factor G	Sorghum bicolor	gi|242076604	5.42	5.58	85.0	85.3	24	128	1∶2.60	14
S28	small ribosomal protein 4	*Bucklandiella crispipila*	gi|160415804	10.31	7.12	52.8	21.7	31	77	1∶9.71	7
Protein fate											
P36	RuBisCO large subunit-binding protein subunit alpha	*Zea mays*	gi|226493235	5.2	5.44	63.5	61.4	23	77	1∶4.26	11
P40	chaperonin 10 Kd subunit	*Zea mays*	gi|195643284	7.74	5.76	26.4	25.8	42	74	1∶2.18	6
P41	Hsp70 (AA 6 - 651)	*Petunia* x hybrida	gi|20559	5.07	5.58	78.3	71.1	19	75	1∶2.21	9
P54	maize 20S proteasome alpha subunit	*Zea mays*	gi|162463728	6.1	6.71	28.1	27.5	34	80	1∶5.26	8
P58	chaperonin GroEL	*Oryza sativa* Japonica Group	gi|115488160	5.12	5.39	64.5	61.2	24	80	1∶12.76	10
S16	heat shock cognate 70 kDa protein 2	*Zea mays*	gi|293334615	5.13	5.38	79.7	71.5	27	113	1∶1.93	16
S36	acetylornithine deacetylase	*Zea mays*	gi|226491976	5.45	6.11	37.4	49.5	41	132	1∶4.73	14
S41	heat shock protein 40	*Oryza sativa* Japonica Group	gi|115448597	8.93	6.2	29.2	49.6	18	84	1∶3.87	7
S42	hydroxyproline-rich glycoprotein-like protein	*Arabidopsis thaliana*	gi|18402131	9.67	6.26	51.6	62.7	16	81	1∶3.64	9
S49	SOUL heme-binding protein	*Zea mays*	gi|194704038	7.85	6.51	107.2	24.2	31	74	1∶3.30	7
S52	FKBP-type peptidyl-prolyl cis-trans isomerase	*Zea mays*	gi|226505004	9	7.14	26.9	26.8	40	79	1∶3.78	7
Secondary metabolism											
P28	1-aminocyclopropane-1-carboxylate oxidase	*Zea mays*	gi|195613470	5.9	6.43	48.3	40.9	39	78	1∶4.14	11
P30	S-adenosylmethionine synthetase	*Zea mays*	gi|100801534	5.83	6.17	52.7	43.0	29	78	1∶6.01	8
P39	beta-D-glucosidase precursor	*Zea mays*	gi|162464369	6.72	6.68	62.7	64.3	25	130	1∶7.41	16
P55	SAM-dependent methyltransferases	*Zea mays*	gi|223942327	6.71	6.49	64.9	29.4	37	103	1∶2.6	9
P62	spermidine synthase	*Zea mays*	gi|223946537	5.15	5.65	37.5	35.3	27	75	1∶31.15	8
S3	glutamate-1-semialdehyde 2,1-aminomutase	*Zea mays*	gi|195639018	6.13	5.47	37.4	50.2	20	88	3.07∶1	6
S9	probable cinnamyl alcohol dehydrogenase	*Zea mays*	gi|162460804	5.95	6.59	57.2	39.2	29	75	1∶1.76	8
S38	arginine decarboxylase	*Zea mays*	gi|219885457	5.18	5.71	52.9	71.1	21	76	1∶5.96	13
S51	1-aminocyclopropane-1-carboxylate oxidase	*Zea mays*	gi|195613470	5.9	6.37	50.9	40.9	43	161	1∶1.83	15
Unknown											
P15	OSJNBa0067K08.7	*Oryza sativa* Japonica Group	gi|21740778	9.21	6.43	32.5	32.4	16	72	1∶0	6
S17	hypothetical protein LOC100194029	*Zea mays*	gi|212722290	9.11	6.95	37.7	31.9	35	81	2.26∶1	8
S31	Os02g0493300	*Oryza sativa* Japonica Group	gi|115446205	6.34	6.05	56.2	48.8	23	76	1∶7.82	7
S34	Os04g0620200	*Oryza sativa* Japonica Group	gi|115460610	8.14	5.41	50.1	31.6	29	74	1∶5.36	6
S39	hypothetical protein	*Zea mays*	gi|226507242	6.30	7.1	52.0	33.8	54	114	0∶1	13
S44	predicted protein	*Physcomitrella patens subsp*. patens	gi|168048747	9.58	6.48	26.9	35.1	29	75	1∶3.47	7
S50	hypothetical protein LOC100275466	*Zea mays*	gi|226531001	5.61	5.87	60.5	41.3	18	78	1∶7.88	7
Energy											
P27	fructose-bisphosphate aldolase, cytoplasmic isozyme 1	*Zea mays*	gi|195612198	6.26	6.79	46.4	38.5	36	77	1∶1.78	10
P44	phosphoglycerate kinase	*Zea mays*	gi|223975935	5.21	5.58	45.9	43.2	55	170	1∶2.34	15
P48	fructose-1,6-bisphosphate aldolase cytoplasmic	*Zea mays*	gi|223975775	6.37	6.29	39.9	38.4	19	77	1∶2.33	7
P50	enolase 1	*Zea mays*	gi|162458207	5.2	5.63	58.6	48.3	53	104	1∶3.84	12
P60	phosphogluconate dehydrogenase	*Zea mays*	gi|162463403	6.24	6.69	52.1	53.2	31	82	1∶3.98	13
P63	6-Phosphogluconolactonase	*Zea mays*	gi|224029461	5.08	5.56	33.5	29.0	27	78	1∶32.78	8
S4	F1-ATP synthase, beta subunit, mitochondrion	Sorghum bicolor	gi|4388533	5.25	5.49	65.7	49.2	54	175	1∶5.42	19
S11	succinate dehydrogenase flavoprotein subunit, mitochondrial precursor	*Zea mays*	gi|195647178	6.08	6.27	76.6	68.6	34	138	1∶1.78	17
S26	6-phosphofructo-2-kinase cytoplasmic	*Zea mays*	gi|2286155	6.15	5.16	23.7	43.5	16	89	1∶2.53	6
S27	glyceraldehyde 3-phosphate dehydrogenase cytoplasmic	*Zea mays*	gi|194700850	6.4	6.82	54.1	32.1	32	78	1∶13.18	6
S40	glyceraldehyde 3-phosphate dehydrogenase cytoplasmic	*Zea mays*	gi|195622314	6.32	7	54.9	30.5	27	74	1∶3.08	7
S46	glyceraldehyde-3-phosphate dehydrogenase A, chloroplastic precursor	*Zea mays*	gi|162461856	7	6.97	54.8	43.2	33	84	1∶2.28	9
S53	glyceraldehyde-3-phosphate dehydrogenase B	*Zea mays*	gi|194688752	5.95	5.84	52.2	47.7	30	80	1∶1.93	10
Cell rescue, defense and virulence											
P6	glutathione S-transferase	*Zea mays*	gi|194703484	4.97	5.51	33.6	27.4	48	85	1∶4.99	9
P13	ascorbate peroxidase cytoplasmic	*Zea mays*	gi|194707280	5.28	5.85	30.9	27.3	42	82	1∶2.27	11
P29	universal stress protein 2	*Gossypium arboretum*	gi|166203459	5.44	6.42	43.5	19.2	28	83	1∶1.58	8
P31	peptide methionine sulfoxide reductase	*Zea mays*	gi|293332177	5.58	5.65	23.1	20.7	30	72	1∶2.47	6
P35	glutathione S-transferase	*Zea mays*	gi|226505920	5.54	6.16	25.3	23.5	65	125	1∶3.17	10
P49	APx1 - Cytosolic ascorbate peroxidase	*Zea mays*	gi|226504576	5.65	7.3	27.5	27.5	53	104	1∶3.05	12
P53	APx1 - Cytosolic ascorbate peroxidase	*Zea mays*	gi|226504576	5.65	6.02	29.1	27.5	52	115	1∶4.38	12
P61	glutathione S-transferase 4	*Zea mays*	gi|162460024	5.77	6.27	26.8	24.7	46	96	1∶3.15	13
S7	early dehydration inducible protein	*Craterostigma plantagineum*	gi|38641280	5.52	6.1	22.3	10.1	31	72	1∶2.84	6
Transcription/cellular communication/signal transduction											
P3	guanine nucleotide-binding protein beta subunit	*Zea mays*	gi|226498574	6.13	6.8	42.2	36.7	44	87	3.85∶1	10
P56	14-3-3 protein	*Zea mays*	gi|194701240	4.8	5.29	33.6	29.4	50	106	1∶11.48	10
P59	RNase	*Vitis vinifera*	gi|147843505	5.63	5.35	40.3	37.3	28	76	1∶15.39	9
P65	RNA-binding protein	*Zea mays*	gi|195655323	4.36	5.28	40.1	8.0	67	97	1∶45.90	7
S2	integrin-linked protein kinase family protein	*Arabidopsis thaliana*	gi|18415205	5.71	6.05	33.8	52.9	19	84	1∶2.06	11
S21	ACI14	*Zea mays*	gi|195619258	6.1	6.89	45.2	35.2	39	128	1∶1.69	12
S30	maturase K	*Boerhavia coccinea*	gi|15340912	9.98	6.96	21.3	32.6	29	80	1∶8.38	6
S35	ACI14	*Zea mays*	gi|195619258	6.1	6.04	46.1	35.2	36	93	0∶1	11
S43	AT5G50010 DNA binding transcription factors	*Arabidopsis thaliana*	gi|227202838	5.44	6.5	28.1	31.3	22	76	1∶3.86	7
S48	component of dynein regulatory complex	*Chlamydomonas reinhardtii*	gi|159478409	8.38	5.49	16.4	54.6	15	80	1∶2.96	9
Cell cycle and transport											
P45	MCM2-related protein	*Arabidopsis thaliana*	gi|1565223	7.27	5.58	42.8	55.1	21	73	1∶3.64	6
S14	Chain A, Crystal Structure of The Complex Between Ferredoxin and Ferredoxin-NADP+ Reductase	*Zea mays*	gi|13096165	7.01	7.19	45.3	35.6	37	121	15.29∶1	13
S20	Ferredoxin	*Zea mays*	gi|162458489	7.55	6.15	43.5	41.2	51	157	11.10∶1	16
S22	thioredoxin h homolog2	*Zea mays*	gi|162459002	6.19	6.73	17.2	13.2	53	76	0∶1	6
S37	thioredoxin H-type	*Zea mays*	gi|195615214	6.19	6.32	54.5	13.2	53	84	1∶4.54	8
S47	NADPH-dependent FMN reductase	*Zea mays*	gi|194704678	5.95	6.35	31.1	21.6	43	72	1∶3.20	9

a: Assigned spot numbers, as indicated in Figure 2.

b: Names of proteins identified by MALDI-TOF/MS.

c: Plant species containing the matching peptides.

d: Database accession numbers from NCBI nr.

e: Theoretical pI of the identified proteins. Theoretical values were retrieved from the protein database.

f: Experimental pI of the identified proteins. The experimental values were calculated using PDQuest Software (Version 7.2).

g: Theoretical masses (kDa) of the identified proteins. Theoretical values were retrieved from the protein database.

h: Experimental masses (kDa) of the identified proteins. The experimental values were calculated using PDQuest Software (Version 7.2).

i: Amino acid sequence coverage for the identified proteins.

k: Mascot score obtained after searching against the NCBI nr database.

m: Specificity indicates the ratio of accumulation of a particular protein from shoots of maize plants under +P *versus* -P conditions.

n: Number of peptides identified for predicted protein.

### Several proteins were up-regulated by low phosphorus stress

Phosphorus deficiency regulates the accumulation of several groups of proteins (Table 6). This includes an increased abundance of proteins related to energy metabolism, such as 6-phosphogluconolactonase (P63), phosphogluconate dehydrogenase (P60), phosphoglycerate kinase (P44), fructose-1,6-bisphosphate/aldolase (P27, P48), glyceraldehyde-3-phosphate dehydrogenase (S27, S40, S46, S53), enolase1 (P50), succinate dehydrogenase of mitochondria (S11) and succinyl-CoA synthetase (P33). These proteins are involved in the pentose phosphate pathway (PPP), glycolysis (EMP) and the tricarboxylic acid cycle (TCA) pathways. Energy metabolism is well known to produce energy and NADPH, and the increased accumulation of proteins involved in the PPP, EMP and TCA pathways is likely to be very important for the control of energy metabolism in maize by increasing the supply of ATP, NADH and NADPH.

The second group of proteins affected by phosphorus starvation is proteins involved in transcription and signal transduction, including 14-3-3 protein (P56), RNA-binding protein (P65) and ACI14 (S21, S35). These proteins participate in phosphorus starvation responses by regulating several metabolic pathways through the phosphorylation of associated proteins. Phosphorus starvation regulated the accumulation of stress- and defense- related proteins, such as the molecular chaperones HSP70 (P41, S16), Cpn-60α subunit of RuBisCO large subunit-binding protein (P36), 10 KDa of Cpn-21 (P40) and FKBP-type peptidyl-prolyl cis-trans isomerase family protein (S52). Molecular chaperones may play an important role in plant adaption to phosphorus deficiency by preventing uncontrolled protein aggregation and promoting the assembly of many polypeptides into their active conformations. Phosphorus starvation induced the accumulation of L-ascorbate peroxidase 1 (P13, P49, P53), GDP-mannose-3′, 5′-epimerase (GME P46, S45) and glutathione-s-transferases (GSTs P6, P35), which likely protect maize from damage caused by abiotic stresses through scavenging ROS.

Proteins involved in ethylene biosynthesis, such as 1-aminocyclopropane-1-carboxylate oxidase (ACC oxidase P28, S51), S-adenosylmethionine synthetase (P30) and adenosylhomocysteinase (P51), also accumulated during phosphorus starvation. S-adenosylmethionine synthetase (P30), arginine decarboxylase (ADC S38) and spermidine synthase (SPDS P62), which are involved in polyamine biosynthetic pathways, were up-regulated during phosphorus starvation. Phosphorus starvation may affect ethylene and polyamine metabolism. The current study showed that phosphorus starvation increased the level of serine hydroxymethyltransferase (S32), which is involved in photorespiration. The increased expression of UDP-sulfoquinovose synthase (P57, S29) during phosphorus starvation suggests that sulfoquinovosyl diacylglycerol (SQDG) might replace certain membrane phospholipids. The changes in the abundance of these proteins in response to phosphorus starvation demonstrate that maize has an increased capacity for internal Pi recycle under phosphorus deficiency conditions.

### Proteins down-regulated by phosphorus deficiency

**Figure 3:**
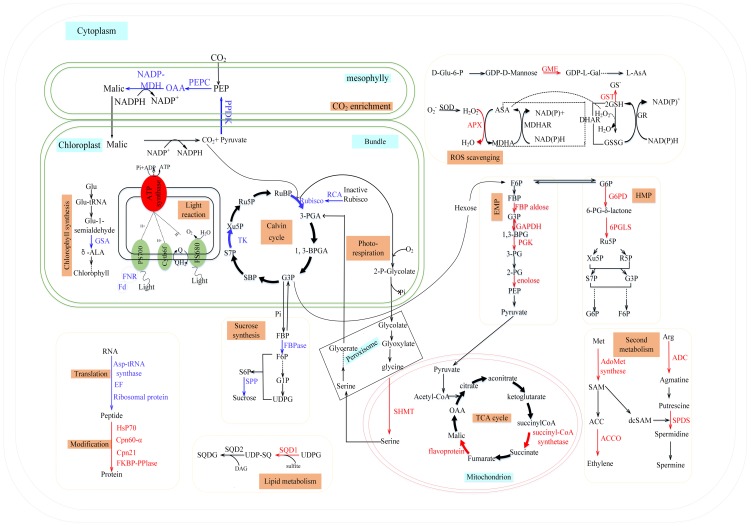
Schematic model of systematic phosphorus tolerance mechanisms in maize. The proteins identified by MALDI-TOF/MS were characterized into subcellular metabolic pathways. Protein expression patterns were indicated by marking protein names and arrows in red (increased expression) or blue (decreased expression). FNR: ferredoxin-NADP reductase; Fd: ferredoxin; ACC: 1-aminocyclopropane-1-carboxylate; ACCO: 1-aminocyclopropane-1-carboxylate oxidase; ADC: arginine decarboxylase; AdoMetsynthase: S-adenosylmethionine synthetase; ADP: adenosine diphosphate; ALA: 5-aminolevulinic acid; APX: ascorbate peroxidase; Arg: arginine; ASA: ascorbic acid; ASP: aspartic acid; ATP: adenosine triphosphate; 1,3-BPGA: 1,3-bisphosphoglycerate; 1,3-BPG: 1,3-bisphosphoglycerate; Cpn21: 10 KD subunit of Chaperonin 21; Cpn60: RuBisCO subunit binding-protein alpha subunit precursor; DAG: diacyl glycerol; dcSAM: S-adenosylmethionine decarboxylation; D-Glu6P: D-Glucose 6-phosphate; DHAR: dehydroascorbate reductase; EF: elongation factors; EMP: Embden-Meyerhof-Parnas pathway; FBP: fructose-1,6-bisphosphate; FBPaldose: fructose-1,6-bisphosphate aldose; FBPase: fructose-1,6-bisphosphatase; FKBP-PPlase: FK506 binding protein peptidyl-prolylisomerases; F6P: fructose-6-phosphate; GAPDH: glyceraldehyde-3-phosphate dehydrogenase; GDP-L-Gal: GDP-L-galactose; Glu: glutamic; GME: GDP-D-mannose-3,5-epimerase; G1P: glucose-1-phosphate; G3P: Glyceraldehyde-3-phosphate; G6P: glucose6-phosphate; G6PD: glucose-6-phosphate dehydrogenase; GR: glutathione reductase; GSA: glutamate-1-semialdehyde 2,1-aminomutase; GSSG: oxidized glutathione; GSH: reduced glutathione; GST: glutathione-S-transferase; HMP: hexose-monophophate-pathway; HSP70: heat shock protein 70; L-AsA: L-ascorbic acid; MDHA: monodehydroasorbate; MDHAR: monodehydroasorbate reductase; Met: methionine; NADP-MDH: NADP-Malate dehydrogenase; OAA: oxalacetic acid; PEP: phosphoenolpyruvate; PEPC: phosphoenolpyruvate carboxylase; 2PG: 2-phosphoglycerate; 3PG: 3-phosphoglycerate; 3-PGA: 3-phosphoglycerate; PGK: phosphoglycerate kinase; 6-PG-δ-lactone: 6-phosphoglucono—δ-lactone; 6PGLS: 6-phosphogluconolactonase; PPDK: pyruvate orthophosphate dikinase; Q: coenzyme-Q; QH2: Coenzyme-QH2; RCA: ribulose bisphosphate carboxylase/oxygenase activase; R5P: ribose-5-phosphate; RuBP: Ribulose-1,5-diphosphate; Ru5P: Ribulose-5-phosphate; SAM: s-adenosyl methionine; SBP: sedoheptulose-1,7-diphosphate; SHMT: serine-glycine hydroxymethyltransferase; SOD: superoxide dismutase; S6P: sucrose-6-phosphate; S7P: 7-Phosphosedoheptose; SPDS: spermidine synthase; SPP: sucrose-6-phosphate phosphohydrolase; SQD1: UDP-sulfoquinovose synthase; SQD2: SQDG synthase; SQDG: sulfoquinovosyl diacylglycerol; TCA cycle: Tricarboxylic acid cycle; TK: transketolase; UDPG: uridine diphosphoglucose; UDP-SQ: UDP-sulfoquinovosyl; Xu5P: Xylulose-5-phosphate.

The current results suggest that the reduction in photosynthesis following low phosphorus treatment can be attributed to the reduced levels of (1) proteins in the light-harvesting complex I (LHCI), (2) Calvin cycle enzymes, such as ribulose1,5-bisphosphate carboxylase/oxygenase (RuBisCO P2, P10, P14, P19, P22, P23, P24 and P26), RuBisCO activase (RCA P4) and transketolase (S10), (3) enzymes involved in regulating the concentration of CO_2_, such as NADP-malate dehydrogenase (NADP-MDH S6), pyruvate orthophosphate dikinase (PPDK P12) and phosphoenol pyruvate carboxylase (PEPC P18) and (4) proteins involved in the transfer of light energy in thylakoids, such as chloroplast light-harvesting complex I protein precursor (S25), ferredoxin-nitrite reductase (FNR S1, S13), ferredoxin (S20) and the CF1 beta subunit (P1) and gamma subunit (P7) of ATP synthase (Table 3). Reductions in the levels of these proteins suggest that phosphorus starvation influences the capacity for light absorption and its conversion to chemical energy, carbon assimilation, ribulose-1,5-diphosphate (RuBP) regeneration and ATP production to influence the rate of photosynthesis (Figure 3). The 2-DE analysis showed that the abundance of FBPase (P8), sucrose phosphatase1 (SPP1 S8) and phosphoglucomutase (PGM S12) decreased under low phosphorus treatment, leading to increased starch levels in maize chloroplasts, which is beneficial for Pi utilization. Proteins involved in protein synthesis, including elongation factor (P16), asparaginyl-tRNA synthetase (P17) and ribosomal protein (P5) were also down-regulated following exposure to phosphate deficiency. The changes in their expression patterns suggest that this variation is also a response to phosphorus deficiency.

## Discussion

### Non-stomatal factors that reduce photosynthesis under phosphorus deficiency conditions include changes in the levels of proteins involved in the LHC, the Calvin cycle and CO_2_ fixation in the Kranz anatomy

Given that low-phosphorus stress decreased *Pn* and *Ls* but increased *Ci* (Table 2), our results are consistent with previous studies indicating that non-stomatal factors have significant impacts on photosynthesis during phosphorus deprivation [33], [50], [51]. LHCI and ATP synthase complexes, as well as ferredoxin reductase (FNR) and ferredoxin, are located in the thylakoid membrane and are involved in harvesting light, forming proton gradients, transferring electrons and producing energy. The abundance of these phosphorus-responsive proteins was significantly altered by phosphorus deficiency. FNR (S1, S13) and ferredoxin (S20), which are involved in the photosynthetic electron transport chain, were down-regulated, which led to detrimental effects on NADPH electron transfer. Phosphorus deprivation also decreased the abundance of the CF1 beta subunit (P1) and the gamma subunit (P7) of ATP synthase. By employing the trans-thylakoid membrane proton motive force, ATP synthase can synthesize ATP to power the dark reaction of photosynthesis. Physiological analysis showed that ATP levels in the leaves decreased by 29.32% in plants subjected to phosphorus deficiency stress (Table 1). Hammond *et al.* demonstrated that depressed ATP synthase activity reduces carbon assimilation [52]. Phosphorus deprivation is thought to restrict ATP production by altering the light-reaction component of photosynthesis.

In the current study, proteomic analysis indicated that phosphorus starvation reduces the levels of certain photosynthetic enzymes. Three enzymes involved in the Calvin cycle, which were affected in this manner, including the large subunits of RuBisCO (P2, P10, P14, P19, P22, P23, P24, P26), RCA (P4) and transketolase (S10) (Table 6, Figure 3). RuBisCO plays an important role in carbon fixation in the Calvin cycle [53]. RCA is a new type of chaperone, which can promote and maintain the catalytic activity of RuBisCO [54]. The decrease in RCA levels under phosphorus deficiency might reduce RuBisCO activity, which would reduce the carboxylation efficiency of RuBisCO. Physiological analysis showed that phosphorus deficiency reduces the carboxylase activity of RuBisCO by 65.02%. In higher plants, transketolase is involved in the regeneration of RuBP in plastids. Transketolase activity is a rate-limiting factor in the Calvin cycle [55]. Phosphorus starvation is thought to reduce the rate of RuBP regeneration and inhibit the Calvin cycle.

The site of 18 =  S23oribulokinase (ion.g,proton gradient formtion,CO_2_ fixation in maize is the Kranz anatomy, which comprises mesophyll cells and bundle sheath cells. The Kranz anatomy can increase the concentration of CO_2_ in mesophyll cells [56]. The 2-DE results showed that low availability of phosphorus decreased the abundance of NADP-MDH (S6), PPDK (P12) and PEPC (P18), all of which play important roles in CO_2_ fixation in bundle sheath cells. Reduced levels of these proteins might reduce CO_2_ levels in mesophyll cells. Changes in the levels of most of these CO_2_ assimilation-related enzymes might account for the decline in photosynthesis during phosphorus deprivation.

The reduced abundance of these proteins correlated well with the rates of photochemical reactions, CO_2_ assimilation, the Calvin cycle and RuBP regeneration. The physiological data also indicated that non-stomatal factors might play a key role in the reduction in photosynthesis during phosphorus starvation. The results suggest that the inhibition of plant growth under phosphorus starvation results primarily from the inhibition of photosynthesis.

### Maize leaf cells increase their internal phosphorus utilization efficiency by altering photorespiration, starch synthesis and remodeling lipid membrane composition under phosphorus starvation

Phosphorus deficiency affects the export of triose phosphates from chloroplasts to cytoplasm, where they are converted into starch [16]. Wasaki *et al.* suggested that the accumulation of starch in phosphorus-deficient leaves might help to maintain the balance between the cytoplasm and chloroplasts [12]. Whereas phosphorus deprivation decreased the sucrose contents in leaves, it increased the leaf starch content and decreased the ratio of sucrose to starch (Table 3). Our physiological data indicate that phosphorus deficiency changes the proportional distribution of triose-phosphate-derived carbon to the cellular pools of sucrose and starch. Our 2-DE analysis showed that low-phosphorus treatment decreased the abundance of FBPase (P8), SPP1 (S8) and PGM (S12), as well as significantly decreasing the activity of FBPase, which is involved in sucrose synthesis (Table 3). These three enzymes play important roles in the synthesis of sucrose under phosphorus deficient conditions and affect the distribution of triose phosphate [9], [57]. Our physiological data also indicated that phosphorus deficiency increased the starch levels while decreasing the sucrose levels (Table 3). These results suggest that the accumulation of starch in maize chloroplasts and the ability to restrict sucrose production are adaptations that enable leaves to maintain Pi levels under low phosphorus stress.

Upon phosphorus starvation, the ubiquitous phospholipids in the photosynthetic membranes of higher plants are replaced by specific nonphosphorous lipids, such as SQDG. The function of SQDG under phosphate-limited growth conditions is highly correlated with the regulation of other plant glycerolipid biosynthetic pathways [58]. Genes encoding enzymes involved in sulfolipid synthesis, such as UDP-SQ synthase and SQDG synthase, are up-regulated during phosphorus starvation [59]. SQDG is associated with several protein complexes in photosynthetic membranes, such as chloroplast CF_0_-CF_1_ of ATPase, LHCII-apoproteins and native D1/D2 heterodimers [60]. In the current study, we observed an increase in the abundance of UDP-SQ synthase (SQD1 P57, S29) during phosphorus starvation, which may increase the production of UDP-SQ. UDP-SQ releases SQ for the production of SQDG. Consequently, the results suggest that UDP-sulfoquinovose synthase plays an important role in increasing the use of cellular phosphorus during phosphorus starvation.

Photorespiration can ameliorate phosphorus starvation and reduce photo-inhibition by consuming the surplus energy produced by the photosynthetic pathway and accelerating the recycling of phosphorus, which reduces the impact of low phosphorus stress [61]. Our 2-DE analysis showed that the levels of serine hydroxymethyltransferase (S32), which is involved in photorespiration, increased under low phosphorus conditions [62], [63]. The physiological data demonstrate that photorespiration increased by 57.75% under low phosphorus conditions (Table 3). These results suggest that maize intensifies photorespiration to facilitate Pi recycling and to alleviate restrictions on photosynthesis caused by phosphorus starvation. All of the above results suggest that maize increases internal phosphorus efficiency by activating alterative pathways to metabolize carbon and membrane lipids.

### Increased accumulation of antioxidant enzymes and small antioxidants may help plants avoid severe damage caused by increased ROS production under phosphorus stress

The results of the fluorescence experiment showed that the amount of light captured for the photochemical reactions decreased in PS II (*P*) by phosphorus deprivation, but the excess energy (*Ex*) in PS II increased (Table 5). The accumulation of *Ex* may increase the reactive oxygen species (ROS) content [64]. The physiological data showed that O_2_^–^ production, the level of ion-leakage, and the H_2_O_2_ and MDA content increased in maize during phosphorus starvation (Table 4).

Previous studies have shown that the Ascorbate-Glutathione cycle is the most important anti-oxidation metabolic pathway in chloroplasts, the cytosol and mitochondria [65]. The 2-DE results revealed that phosphorus deprivation increased the level of APX (P13, P49, P53), which is involved in the Ascorbate-Glutathione cycle, as well as GSTs (P6, P35) with glutathione peroxidase activity, which can reduce H_2_O_2_ to H_2_O to protect plants from oxidative damage under abiotic stress [66]. GME (P46, S45) plays a key role in the synthesis of ASA [67], which can scavenge ROS and protect lipids from oxidation. In addition, peptide methionine sulfoxide reductase (P31) accumulates under phosphorus deprivation to help protect cells from oxidization (by reducing methionine sulfoxide to methionine) and to ensure protein activity [68]. The changes in antioxidative protein levels suggest that phosphorus deprivation causes maize to intensify its oxygen-scavenging system to clear ROS rapidly and to maintain the balance of ROS in order to defend the plant against oxidative damage. The physiological data showed that ASA levels and APX activity increased significantly under phosphorus deficiency (Table 4). Therefore, multiple ROS-scavenging mechanisms enable maize leaves to cope with moderate phosphorus deficiency. These mechanisms include increased activity of the Ascorbate-Glutathione cycle and increased synthesis of the low-molecular-weight antioxidant ASA and several other peroxiredoxins, such as the peptide methionine sulfoxide reductase.

### Accumulation of several proteins involved in secondary metabolism may help plants to regulate phosphorus-induced metabolic reaction

S-adenosylmethionine (SAM) is not only a carrier of the methyl group required for DNA and RNA modification, but it is also more generally involved in the transfer of methyl groups to many structural components of plants, acting as the precursor for the synthesis of lignin, pectin and the methylester of polygalacturonic acid [69], [70]. The level of s-adenosylmethionine synthetase, which is indispensable for the SAM cycle, increased significantly under phosphorus-deficient conditions, suggesting that this enzyme is involved in the responses of maize leaves to phosphorus starvation.

We found that the levels of ACC oxidase (P28, S51) and SAM synthetase (P30) increased during phosphorus deficiency. Both 1-aminocyclopropane-1-carboxylate oxidase (P28, S51) and SAM synthetase (P30, P55) play key roles in ethylene synthesis. Phosphorus deficiency is thought to trigger the switch from primary metabolism to secondary metabolism, such as the accumulation of hormones and lignin [71]. Ethylene is involved in plant adaptation to low phosphorus stress and the regulation of plant growth and development [72]. Our results suggest that the ethylene signaling pathway is involved in the response to phosphorus starvation.

Polyamines (PAs) are small, aliphatic amines that are ubiquitous in all living organisms. Previous studies have shown that polyamines play important roles in morphogenesis, growth, embryogenesis, organ development, leaf senescence and biotic and abiotic stress responses [73]. The current study demonstrated that phosphorus starvation increases the levels of S-adenosylmethionine synthetase (P30), ADC (S38) and SPDS (P62), which are involved in PA biosynthesis. Given the close correlation between PA homeostasis and the response to phosphorus deprivation, we postulate that low-phosphorus stress increases the biosynthesis of spermidine.

Since SAM is the common precursor of both polyamines and ethylene, it remains to be established how SAM is allocated for the synthesis of polyamines or ethylene.

## Conclusions

Previous proteomic analyses have focused on the responses of root and suspension cell cultures, rather than leaves, to phosphorus stress. In this study, through 2-DE and MALDI-TOF/MS analysis, we showed that some of the proteins that respond to phosphorus starvation are involved in several major metabolic pathways, such as photosynthesis, carbohydrate metabolism, energy metabolism and secondary metabolism. To understand the functions of proteins involved in the response to phosphorus deficiency, we developed a model of the metabolic network induced by low phosphorus levels in the leaves of maize (Figure 3).

The physiological and 2-DE results showed that Pn and several proteins related to photosynthesis were down-regulated under phosphorus starvation. As a result of the down-regulation of carbon assimilation, the biomass of plants subjected to phosphorus deficiency significantly declined compared with normal growth conditions.

When leaves are exposed to photons in excess of the level required to support CO_2_ fixation, the excess electrons reduce O_2_ to O_2_^. −^ through the Mehler process. The accumulation of ROS induces the activities of chloroplastic and cytosolic antioxidant enzymes and increases the levels of non-enzymatic materials used by plants to avoid the severe damage caused by increased ROS accumulation under phosphorus stress.

The inorganic phosphorus in the cells is exhausted after the maize is exposed to phosphorus deficiency for a long period of time. The plant intensifies the synthesis of starch and SQDG to increase the utilization of cellular phosphorus, which partially relieves the effects of phosphorus starvation. Moreover, the increased photorespiration rate also releases internal phosphorus, which is also used to alleviate phosphorus starvation.

Several proteins among the 116 identified proteins play important roles in linking metabolic adaptations to the regulation of internal phosphorus homeostasis in the plant. These proteins belong to many functional categories and are involved in multiple metabolic and signaling pathways. These results lay the foundation for further studies characterizing maize responses to phosphorus deficient-conditions.

## Supporting Information

Table S1 The identified differentially expressed proteins and the sequences of the identified peptides.
(XLS)

Text S1 MS/MS patterns of sequenced peptides.
(PDF)
