# Supplementary material for: Comparative Proteomic Analyses Provide New Insights into Low Phosphorus Stress Responses in Maize Leaves
Source: PLoS One. 2014 May 23;9(5):e98215. doi: 10.1371/journal.pone.0098215 (PMC4032345; doi:10.1371/journal.pone.0098215)
Supplement: Text S1 — MS/MS patterns of sequenced peptides. (PDF) [file pone.0098215.s002.pdf]

Supporting Information for Table 7: The MS/MS patterns of sequenced petides

Spot number: **P4**

Protein name: **RuBisCO activase1**

gi|162458161

Sequenced peptide: **GLAYDISDDQQDITR**

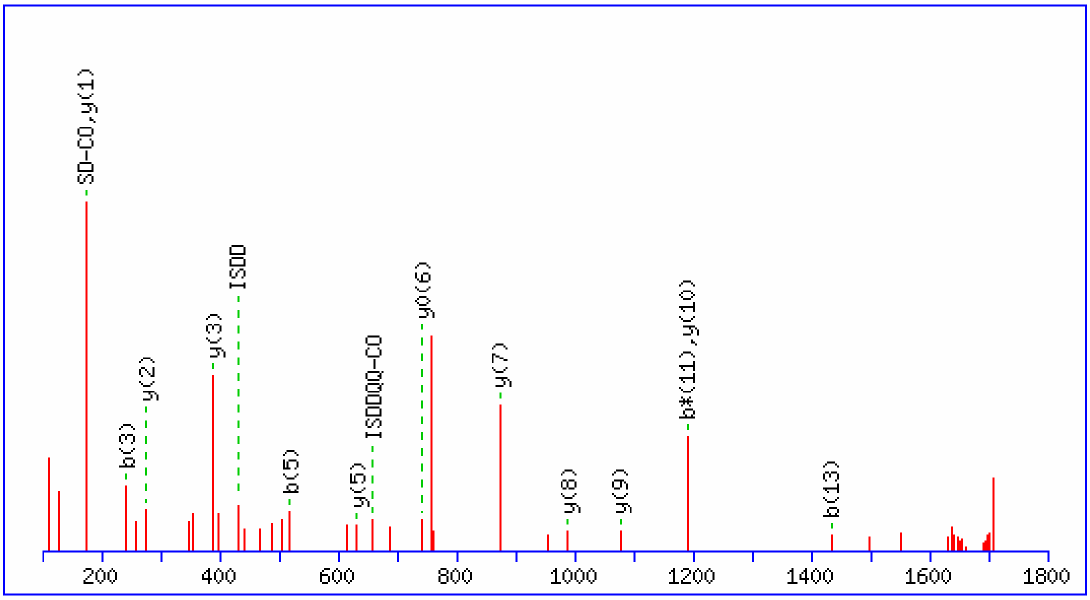

Spot number: **P7**

Protein name: **ATP synthase gamma chain**

gi|226533016

Sequenced peptide: **RPYIPLER**

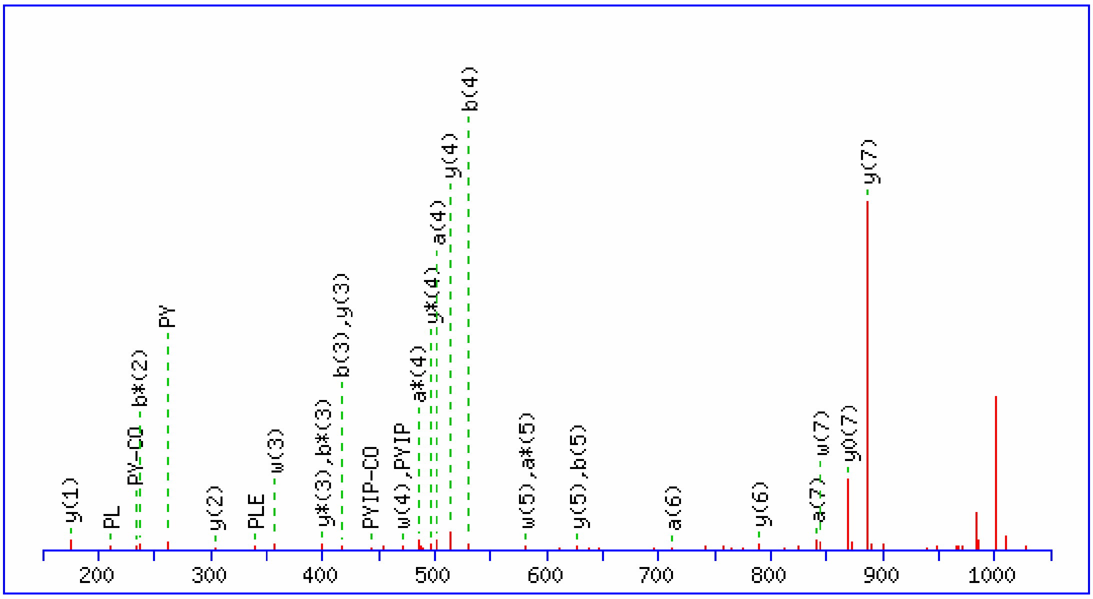

Spot number: **P11**

Protein name: **glutamine synthetase**

gi: |195619796

Sequenced peptide: **VIAEYIWVGGSGIDIR**

HDLHISAYGEGNER

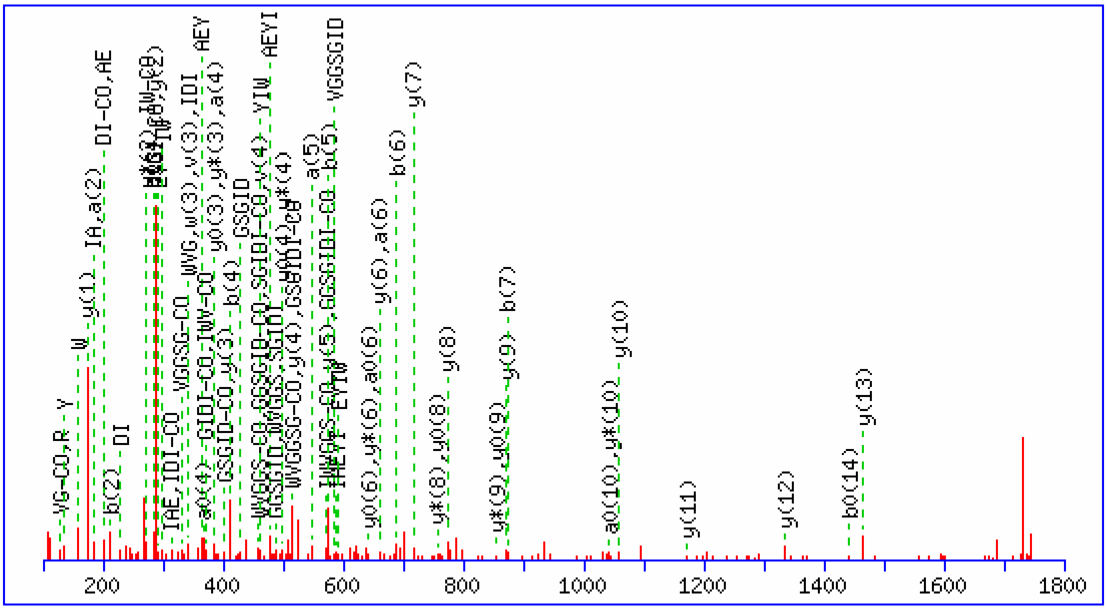

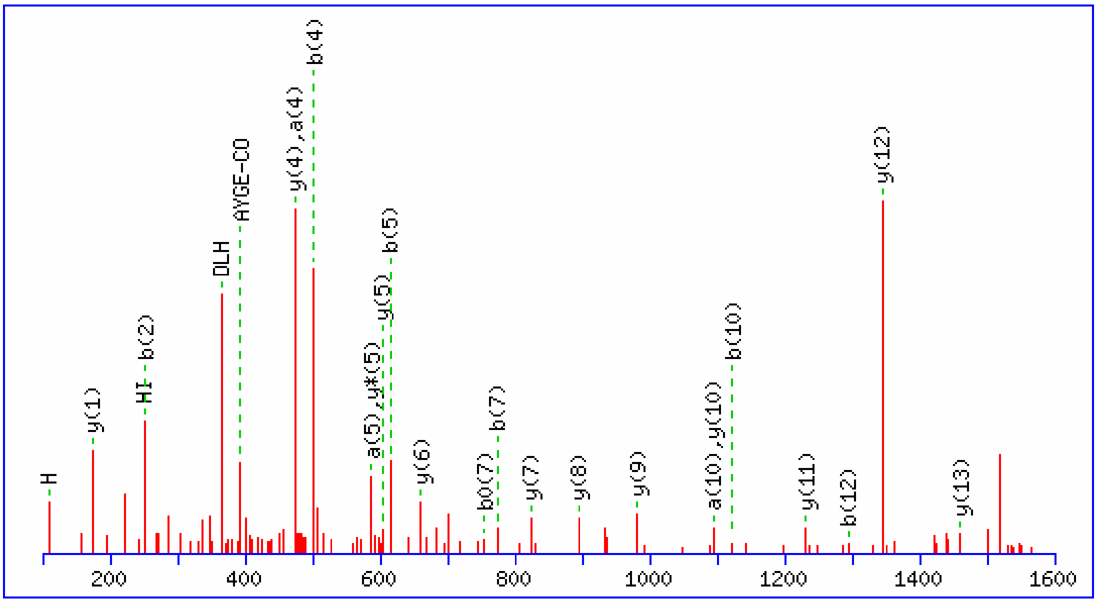

Spot number: **P12**

Protein name: **pyruvate,orthophosphate dikinase**

gi: |168586

Sequenced peptide: **FLDMFGNVVMDIPR**

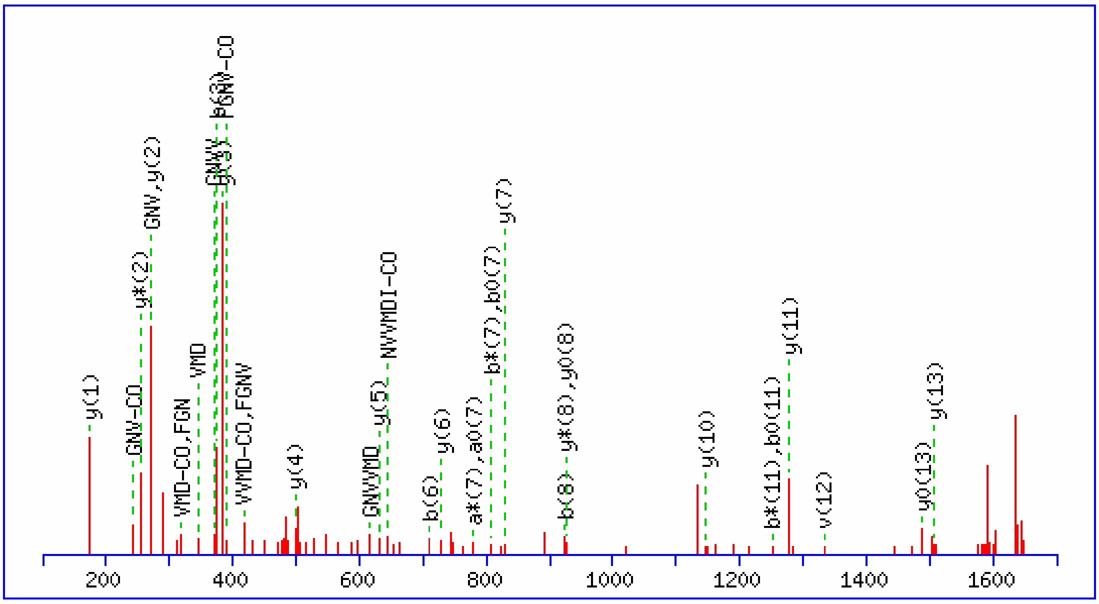

Spot number: **P30**

Protein name: **S-adenosylmethionine synthetase 1**

gi: |194689980

Sequenced peptide: **FVIGGPHGDAGLTGR**

NIGFVSNDVGLDADHCK

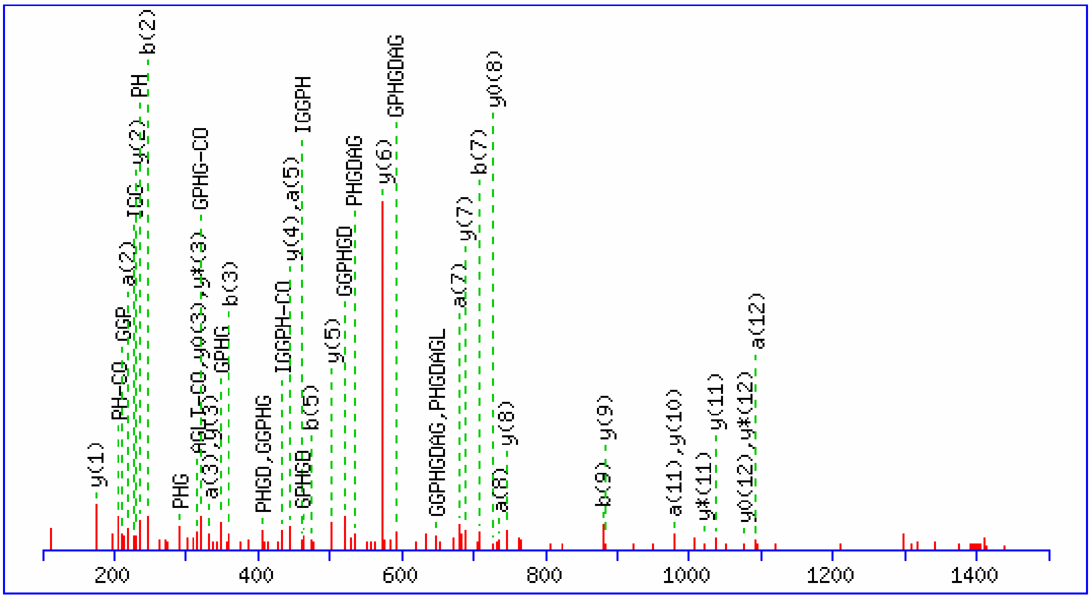

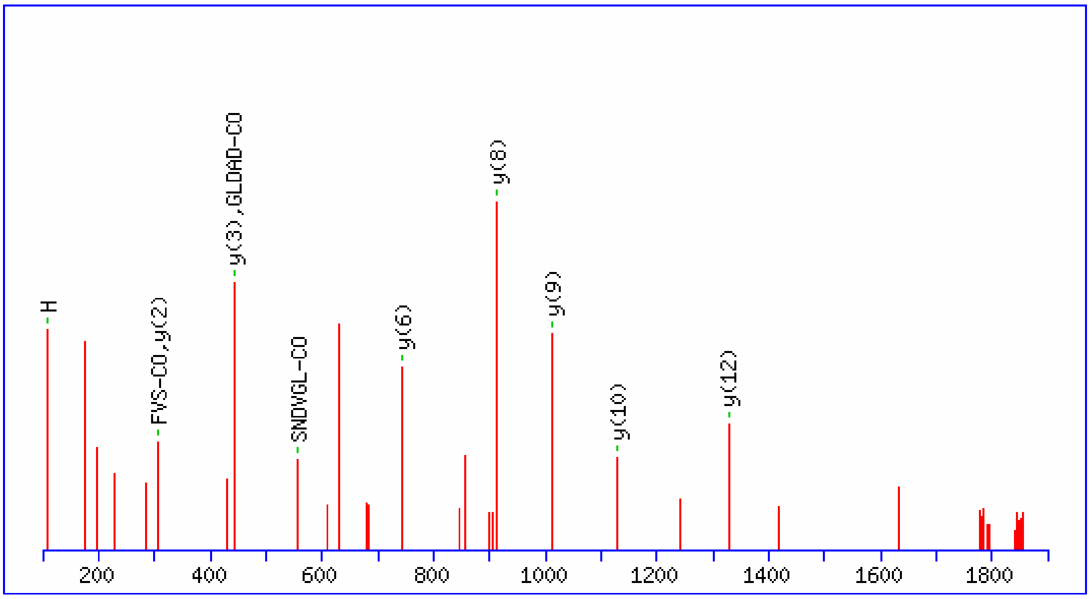

Spot number: **P36**

Protein name: **Rubisco large subunit-binding protein subunit alpha**

gi: **226493235**

Sequenced peptide: **EIAFDQGSR**

AIELADPMENAGASLIR

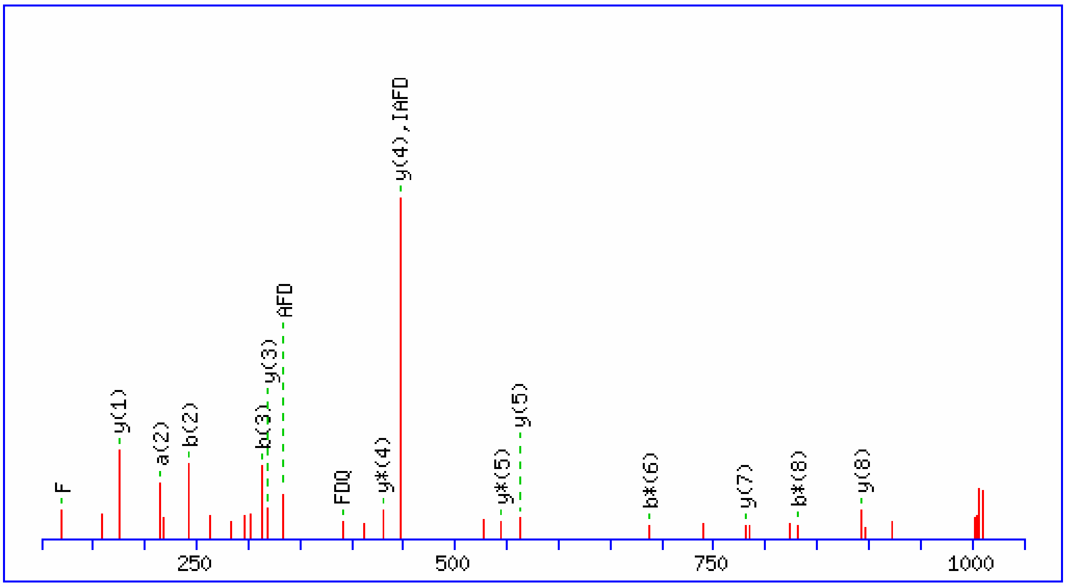

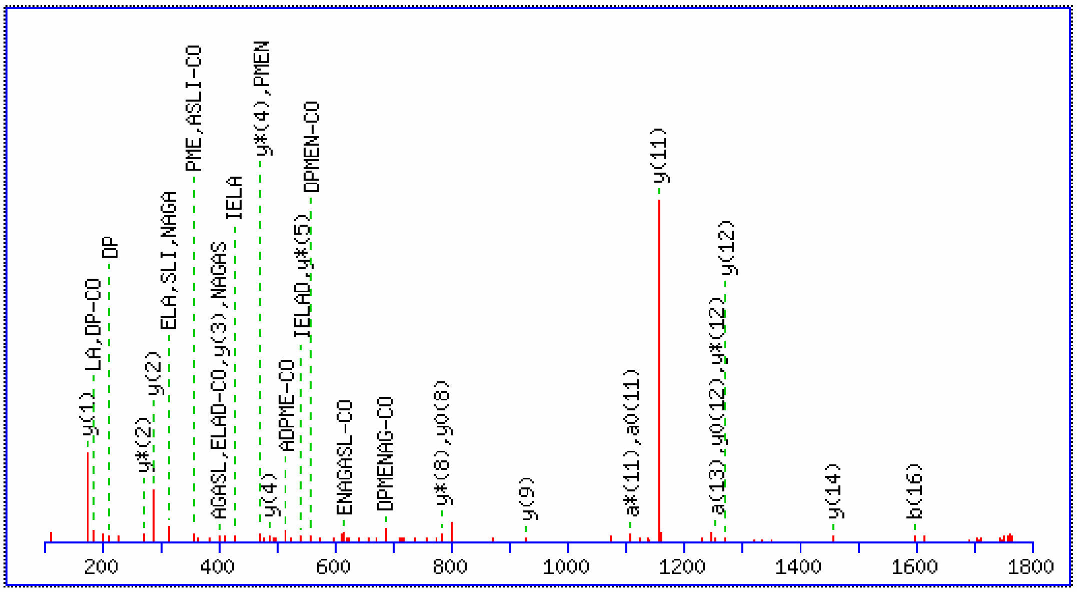

Spot number: **P41**

Protein name: **Hsp70**

gi: 20559

Sequenced peptide: **TTPSYVGFTDTER**

NAVVTVPAYFNDSQR

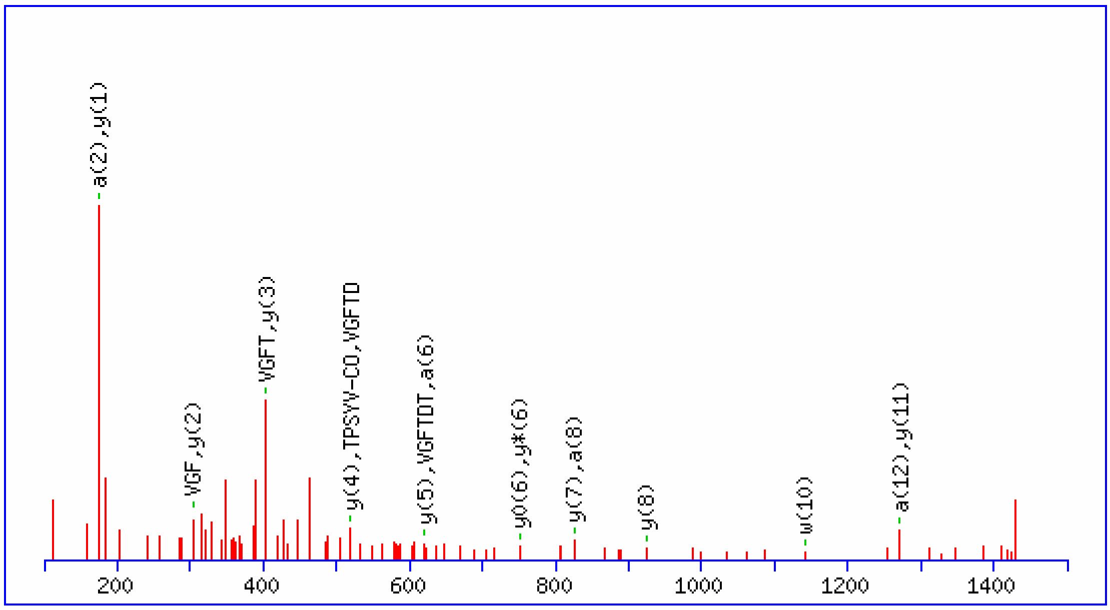

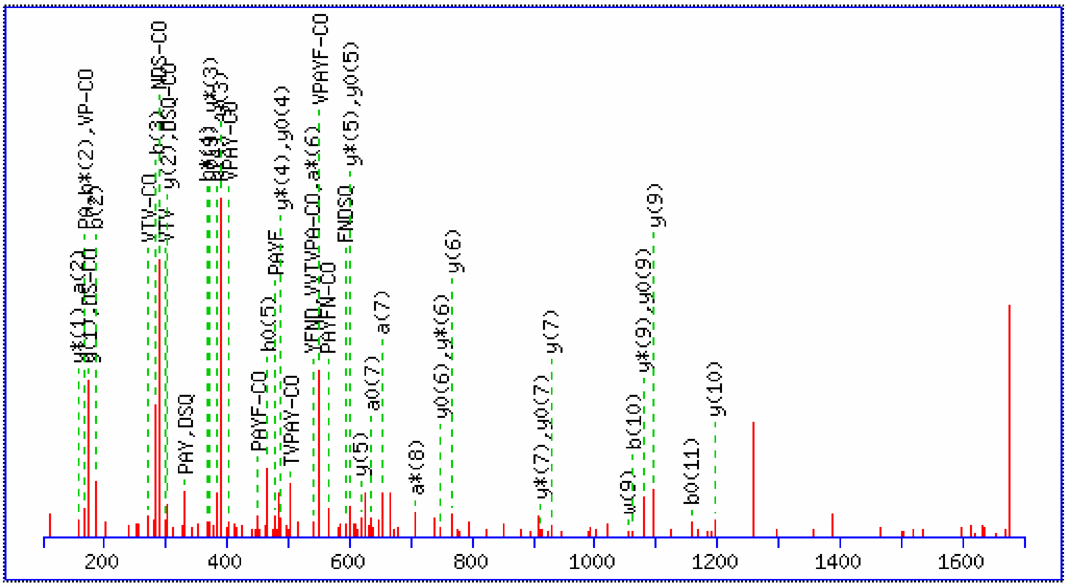

Spot number: **P54**

Protein name: **maize 20S proteasome alpha subunit**

gi: |162463728

Sequenced peptide: **HITIFSPEGR**

CDPAGHFFGHK

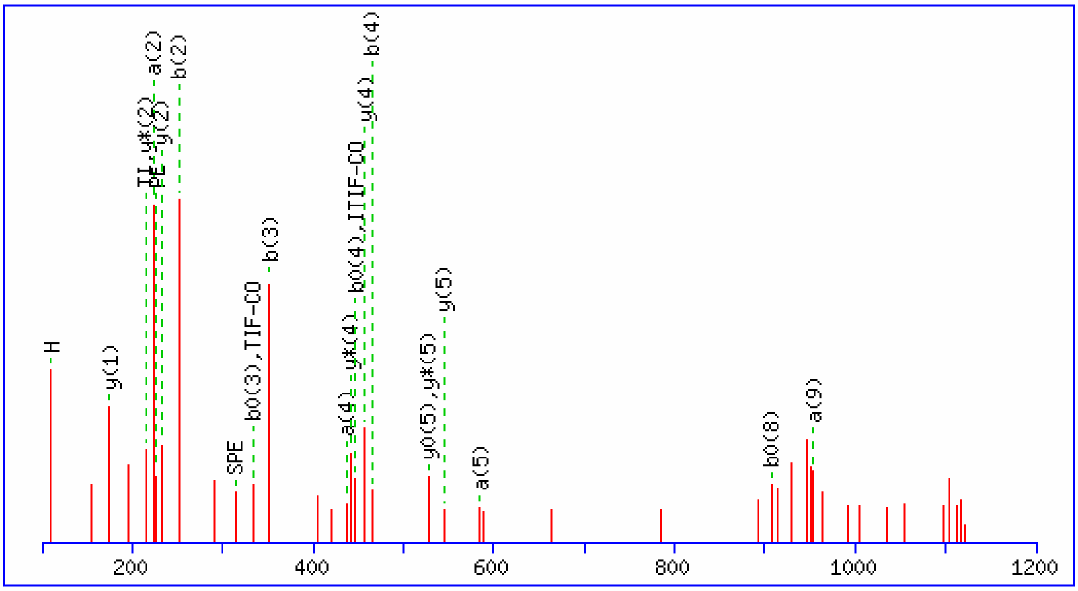

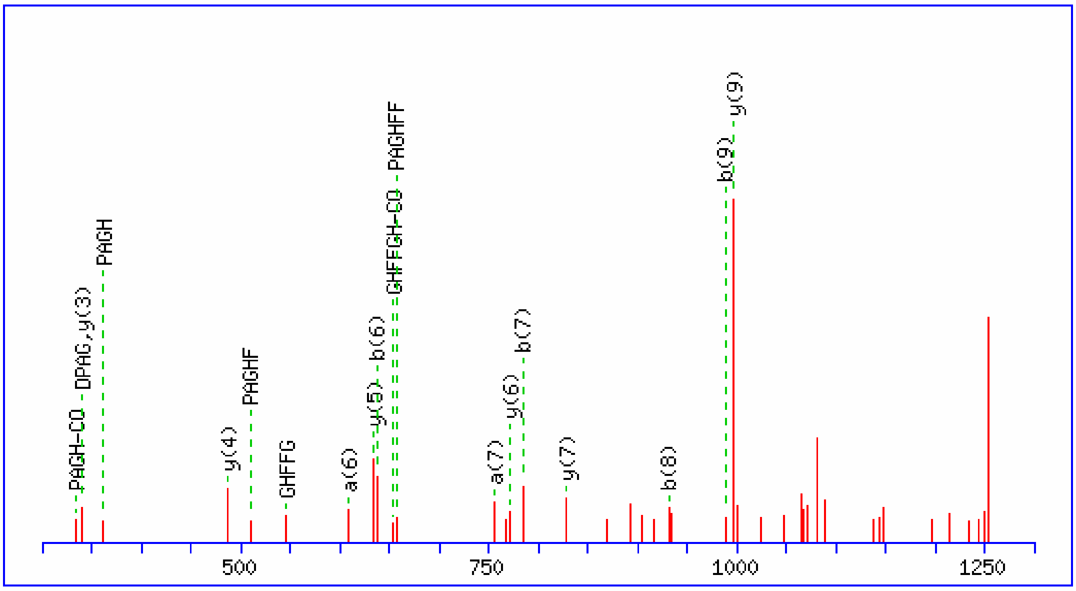

Spot number: **P57**

Protein name: **UDP-sulfoquinovose synthase**

gi: |226530866

Sequenced peptide: **ATDLNQGVVYGVR**

SFEPDAAVHFGEQR

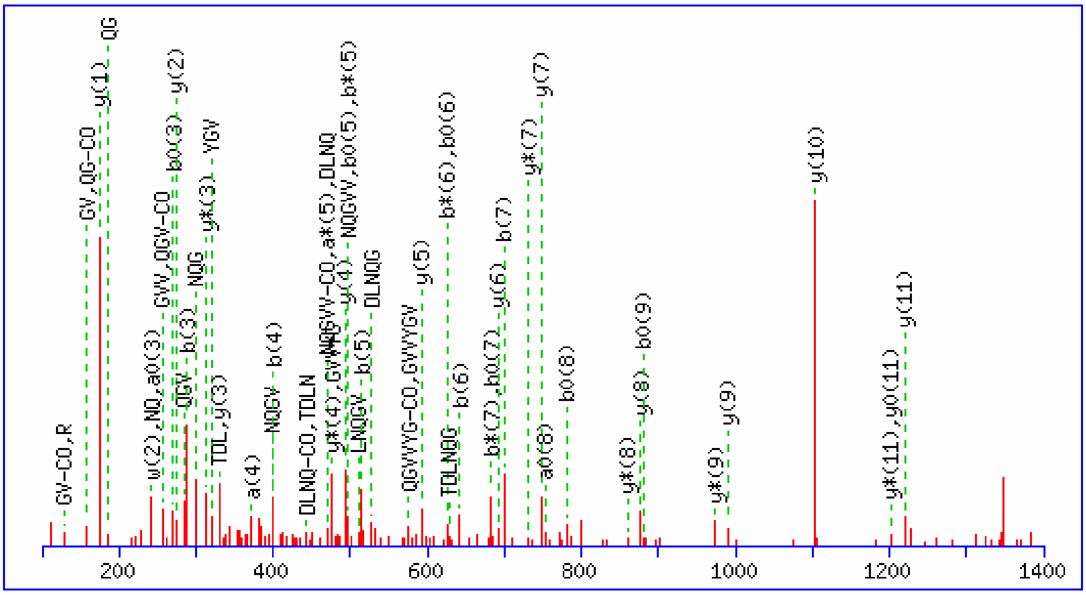

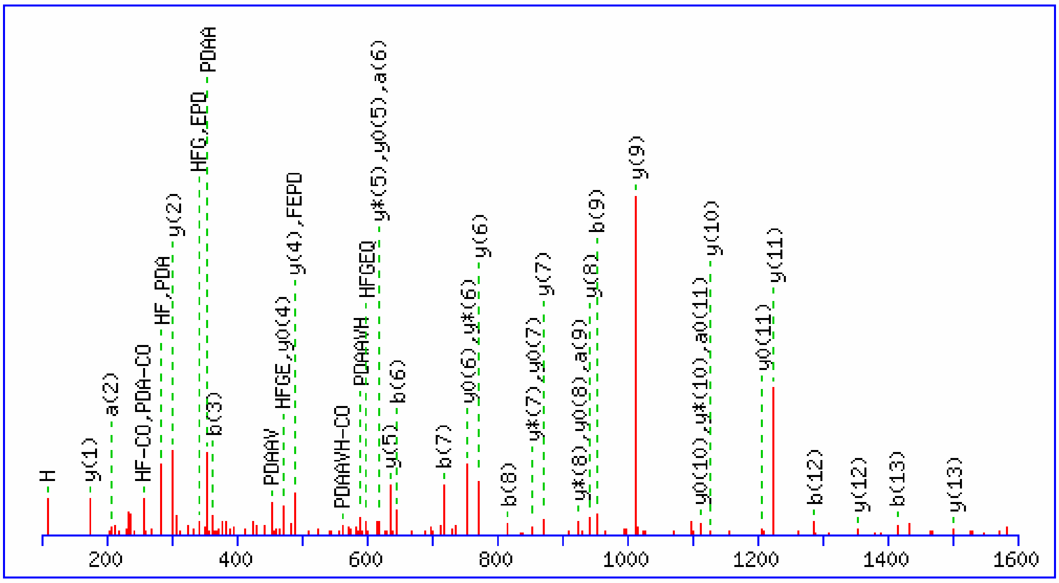

Spot number: **P63**

Protein name: **6-phosphogluconolactonase**

gi: |224029461

Sequenced peptide: **GAFTAVLSGGSLIEALR**

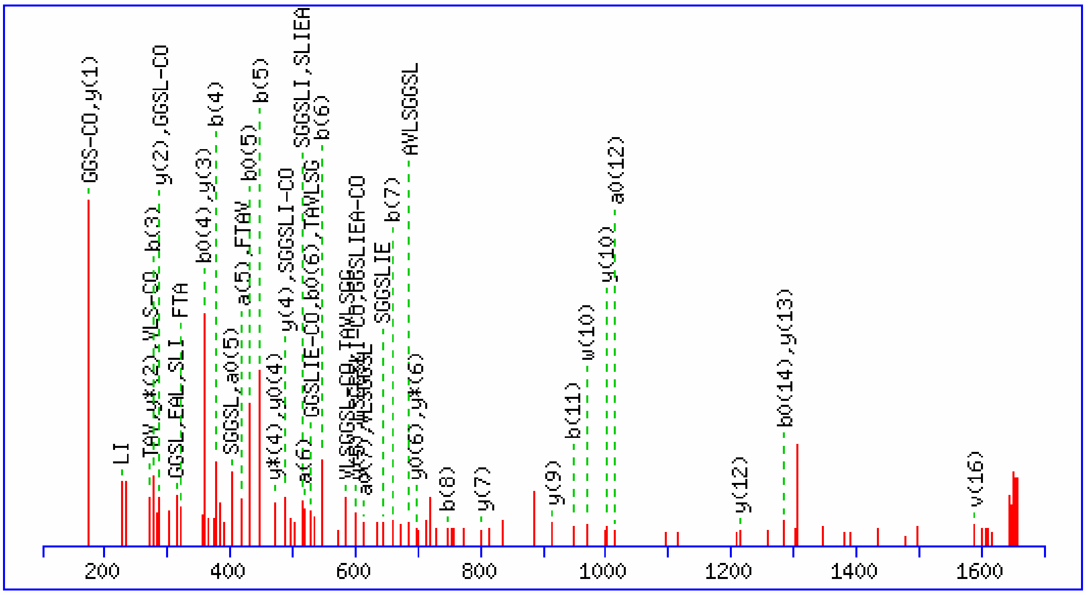

Spot number: **S8**

Protein name: **sucrose-phosphatase1**

gi: |162463022

Sequenced peptide: **CAAGIIQAIGHFK**

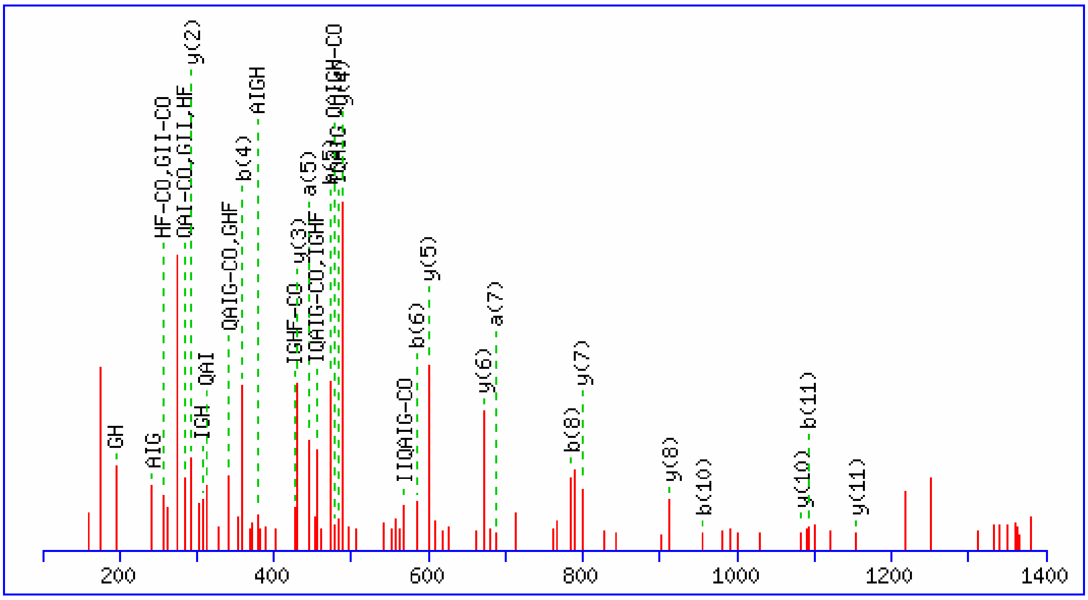

Spot number: **S10**

Protein name: **transketolase**

gi: |194708072

Sequenced peptide: **QKLPHLPGTSIEGVEK**

ISIEAGSTLGWQK

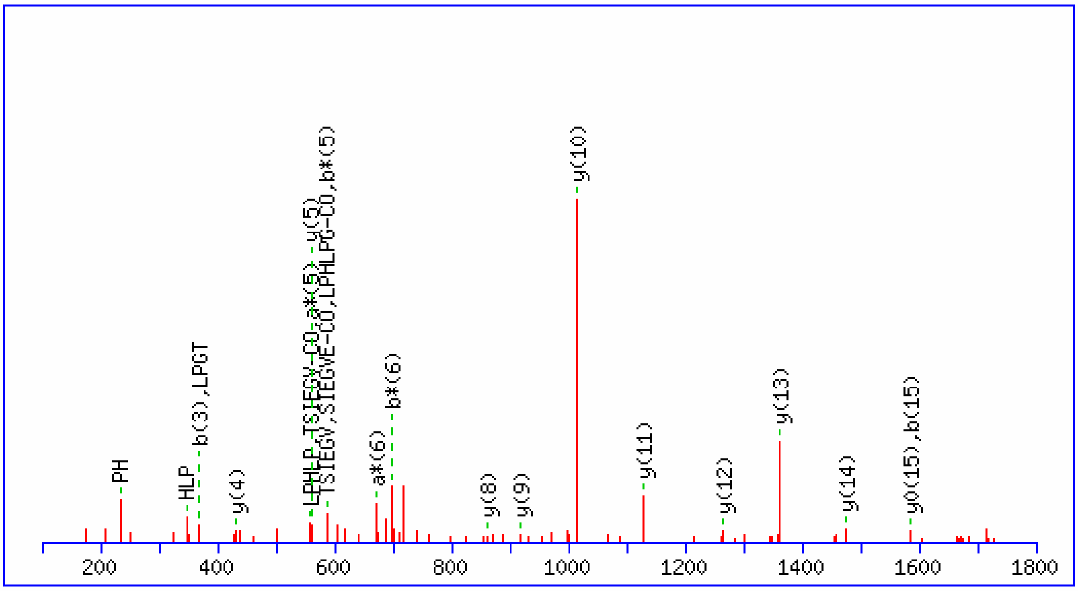

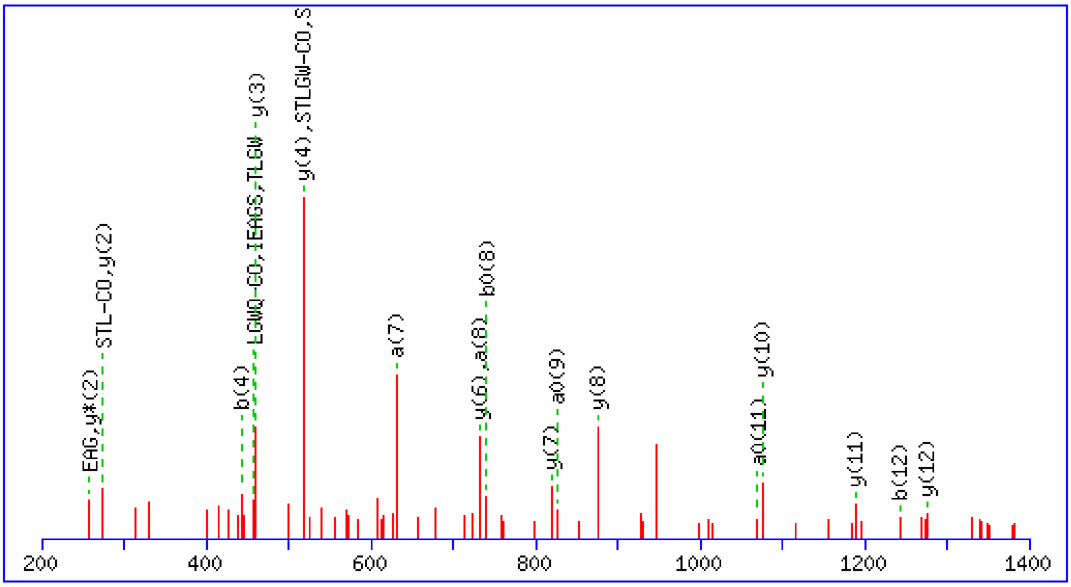

Spot number: **S21**

Protein name: **ACI14**

gi: |195619258

Sequenced peptide: **RVLDFLLALPTR**

VLDFLLALPTR

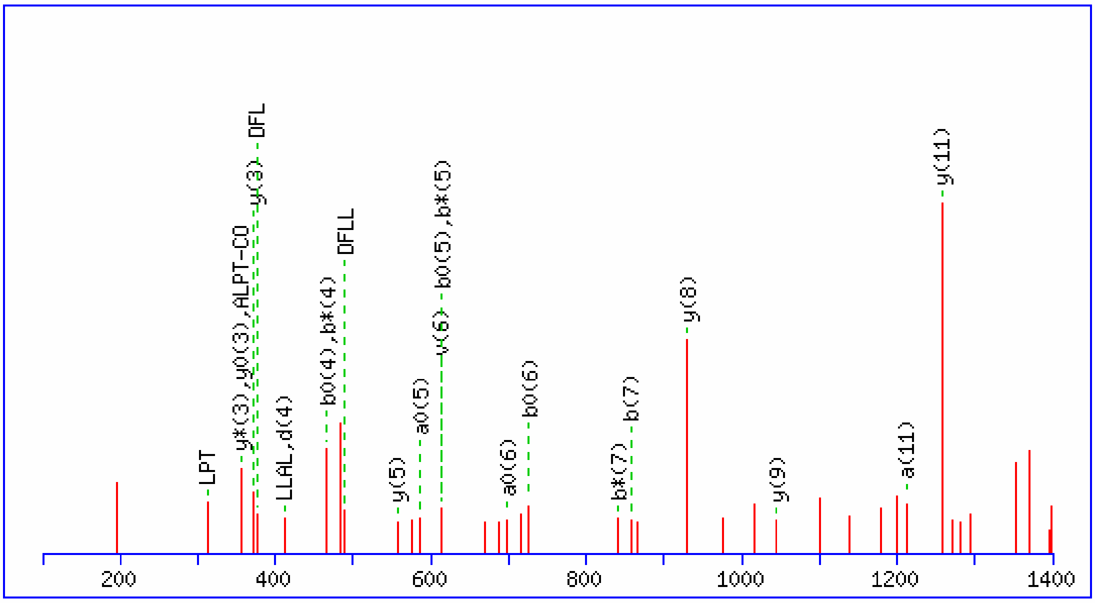

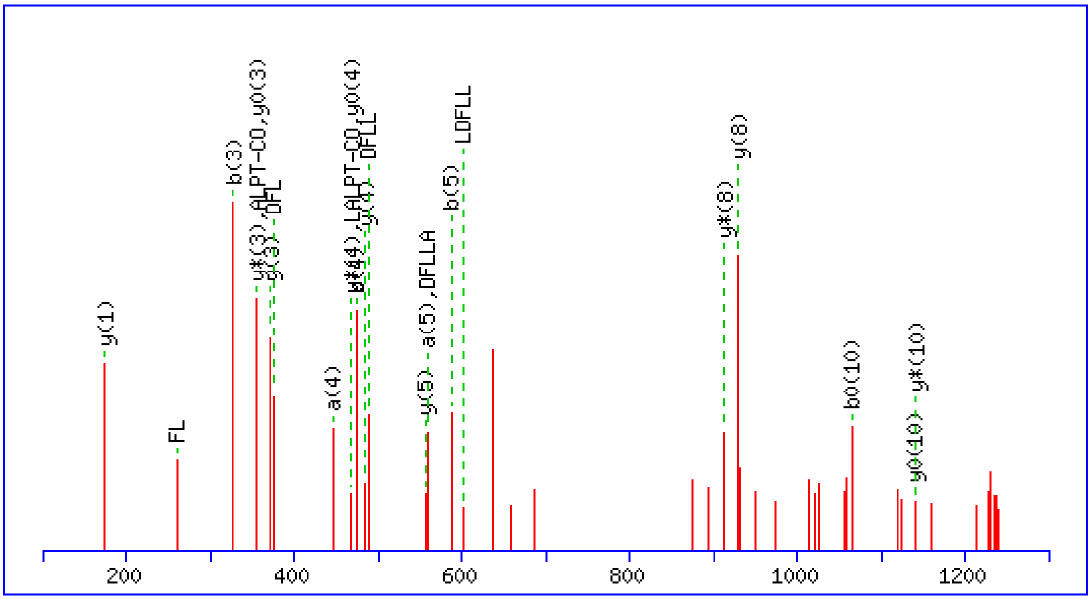

Spot number: **S51**

Protein name: **1-aminocyclopropane-1-carboxylate oxidase**

gi: |195613470

Sequenced peptide: **NFTMSEFYGTFFSR**

AFNELPAHER

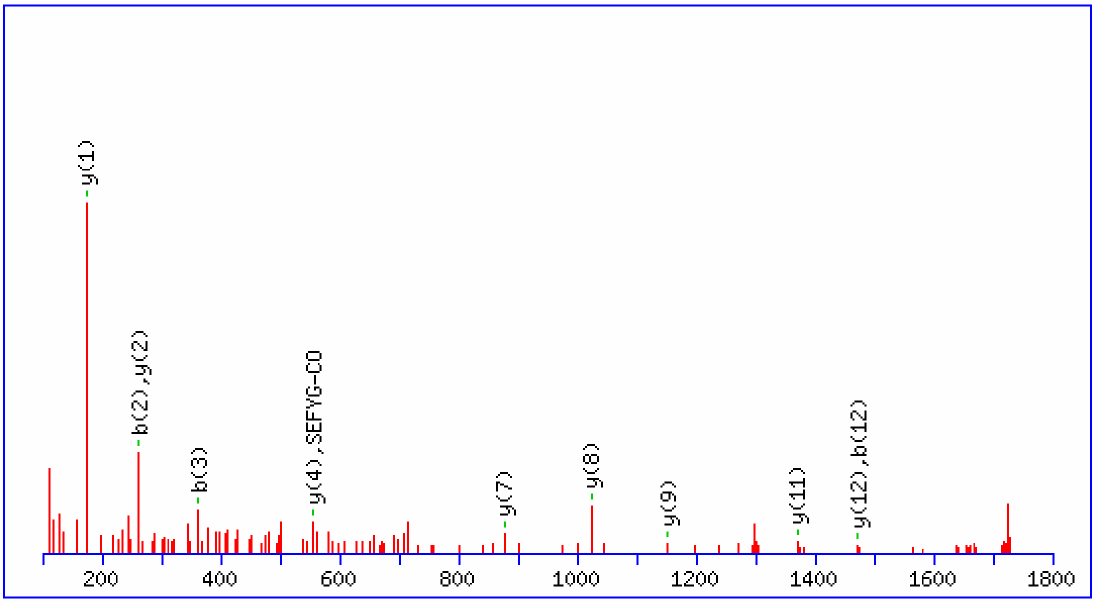

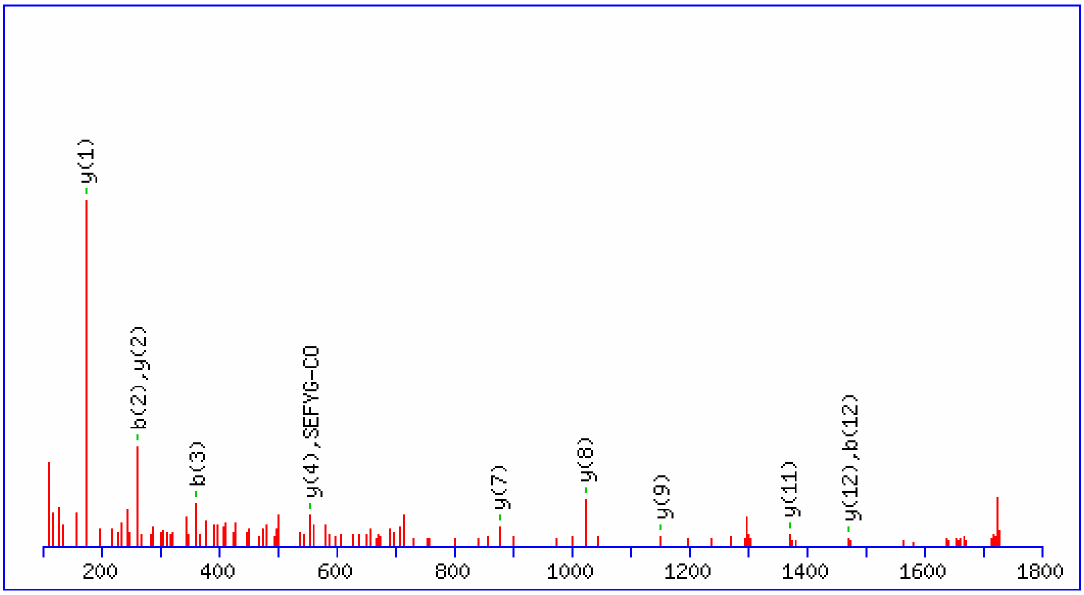
y(1)
b(2),y(2)
b(3)
y(4),SEFYG-CO
y(7)
y(8)
y(9)
y(11)
y(12),b(12)
200
400
600
800
1000
1200
1400
1600
1800
